# The Comprehensive Native Interactome of a Fully Functional Tagged Prion Protein

**DOI:** 10.1371/journal.pone.0004446

**Published:** 2009-02-11

**Authors:** Dorothea Rutishauser, Kirsten D. Mertz, Rita Moos, Erich Brunner, Thomas Rülicke, Anna Maria Calella, Adriano Aguzzi

**Affiliations:** 1 Institute of Neuropathology, University Hospital of Zurich, Zurich, Switzerland; 2 Functional Genomics Center Zurich, Zurich, Switzerland; 3 Center for Model Organism Proteomes, University of Zurich, Zurich, Switzerland; 4 Institute of Laboratory Animal Science and Research Center Biomodels Austria, University of Veterinary Medicine, Vienna, Austria; Swiss Federal Institute of Technology Lausanne, Switzerland

## Abstract

The enumeration of the interaction partners of the cellular prion protein, PrP^C^, may help clarifying its elusive molecular function. Here we added a carboxy proximal myc epitope tag to PrP^C^. When expressed in transgenic mice, PrP_myc_ carried a GPI anchor, was targeted to lipid rafts, and was glycosylated similarly to PrP^C^. PrP_myc_ antagonized the toxicity of truncated PrP, restored prion infectibility of PrP^C^-deficient mice, and was physically incorporated into PrP^Sc^ aggregates, indicating that it possessed all functional characteristics of genuine PrP^C^. We then immunopurified myc epitope-containing protein complexes from PrP_myc_ transgenic mouse brains. Gentle differential elution with epitope-mimetic decapeptides, or a scrambled version thereof, yielded 96 specifically released proteins. Quantitative mass spectrometry with isotope-coded tags identified seven proteins which co-eluted equimolarly with PrP^C^ and may represent component of a multiprotein complex. Selected PrP^C^ interactors were validated using independent methods. Several of these proteins appear to exert functions in axomyelinic maintenance.

## Introduction

The cellular prion protein, PrP^C^, is required for susceptibility to prion infections [1], [2], for prion toxicity [3], and for prion transport within the body [4]. PrP^C^ is a conserved glycoprotein that is anchored to the cell surface through a covalently attached glycosyl phosphatidyl inositol (GPI) residue [5]. PrP^C^ undergoes a complex biogenesis encompassing co-translational secretion into the lumen of the endoplasmic reticulum, cleavage of an N-terminal signal peptide, addition of complex N-linked carbohydrate chains at two sites [6], addition of a preformed GPI anchor at its very C-terminus (Ser^230^), and removal of a C-terminal oligopeptide.

Despite the detailed chemical knowledge described above, the molecular details of the process by which PrP^C^ is converted into a disease-associated homologue, PrP^Sc^, are unclear [7]. Likewise, the chain of events emanating from prion infections and leading to neurodegenerative changes and clinical signs is unknown. Lastly, the physiological function of PrP^C^ is unclear [8]. Most of the above processes may require interactions with proteins other than PrP, yet the nature of such interaction partners is largely unknown. The present study was initiated as an approach to discovering the functionally relevant interaction partners of PrP^C^.

Several diverse approaches have been used in the past to achieve the latter goals. In some instances, however, the techniques employed were not sufficiently sensitive or were fraught with other problems. Classical two-hybrid screens, in which fusion proteins leads to biological readouts in the cytosol of yeast, tend to produce when applied to membrane proteins like PrP^c^. The same holds true for cross-linking experiments, in which proteins resident in the same micro-environment may become linked together even if they do not functionally interact with each others.

In order to avoid the problems described above, and to minimize any interference with the conditions existing in vivo, we isolated native protein complexes containing PrP^C^ and characterized them by mass spectrometry. The addition of epitope tags, for which high-affinity antibodies are available, has proven instrumental for the study of many supramolecular complexes. The engineering of appropriate tags into the proteins of choice yields “molecular handles” through which multi-component complexes can be immunoprecipitated and highly purified. PrP^C^ lends itself to this approach as a particularly attractive bait, as its high-resolution structure is known [9] and thereby allows for the rational design of tags. If the precipitating antibodies are directed against linear, non-conformational epitopes within the tag, epitope-mimetic peptides can release the protein complexes in a highly specific way under non-denaturing conditions. The introduction of a tag is also a promising starting point for identifying functionally relevant complexes since it preserves protein interactions that occur in the same region of an anti-PrP antibody.

GFP-PrP^C^ fusion proteins have proved useful for determining the subcellular distribution and trafficking of normal and mutated prion protein [10], [11], [12]. However, the suitability of GFP to the proteomic approach delineated above is limited. GFP is a bulky, highly structured and rigid tag whose molecular weight exceeds that of PrP^C^. Therefore we reasoned that GFP may distort the composition of any native multiprotein complex that encompasses PrP^C^.

In the present study, we have tagged the C-terminus of mouse PrP^C^ with the human “myc-tag”. The resulting chimaeric protein, termed PrP_myc_, was used to immunoprecipitate and characterize the supramolecular complex containing the prion protein from transgenic mice. Using immunoprecipitation and mass spectrometry, we have identified a set of proteins associated with PrP_myc_. Since the conversion of cellular prion protein PrP^C^ into the proteinase K-resistant isoform PrP^Sc^ is the central pathogenic process in prion diseases, we investigated whether PrP_myc_ can be converted into PrP^Sc^. Our results indicate that C-terminally myc-tagged prions can contribute to prion infectivity and to neurotoxicity. Therefore, myc tagged PrP^Sc^ may also allow for identification of proteins interacting with PrP^Sc^.

## Results

### Transgenic mice expressing C-terminally tagged PrP

**Figure 1:**
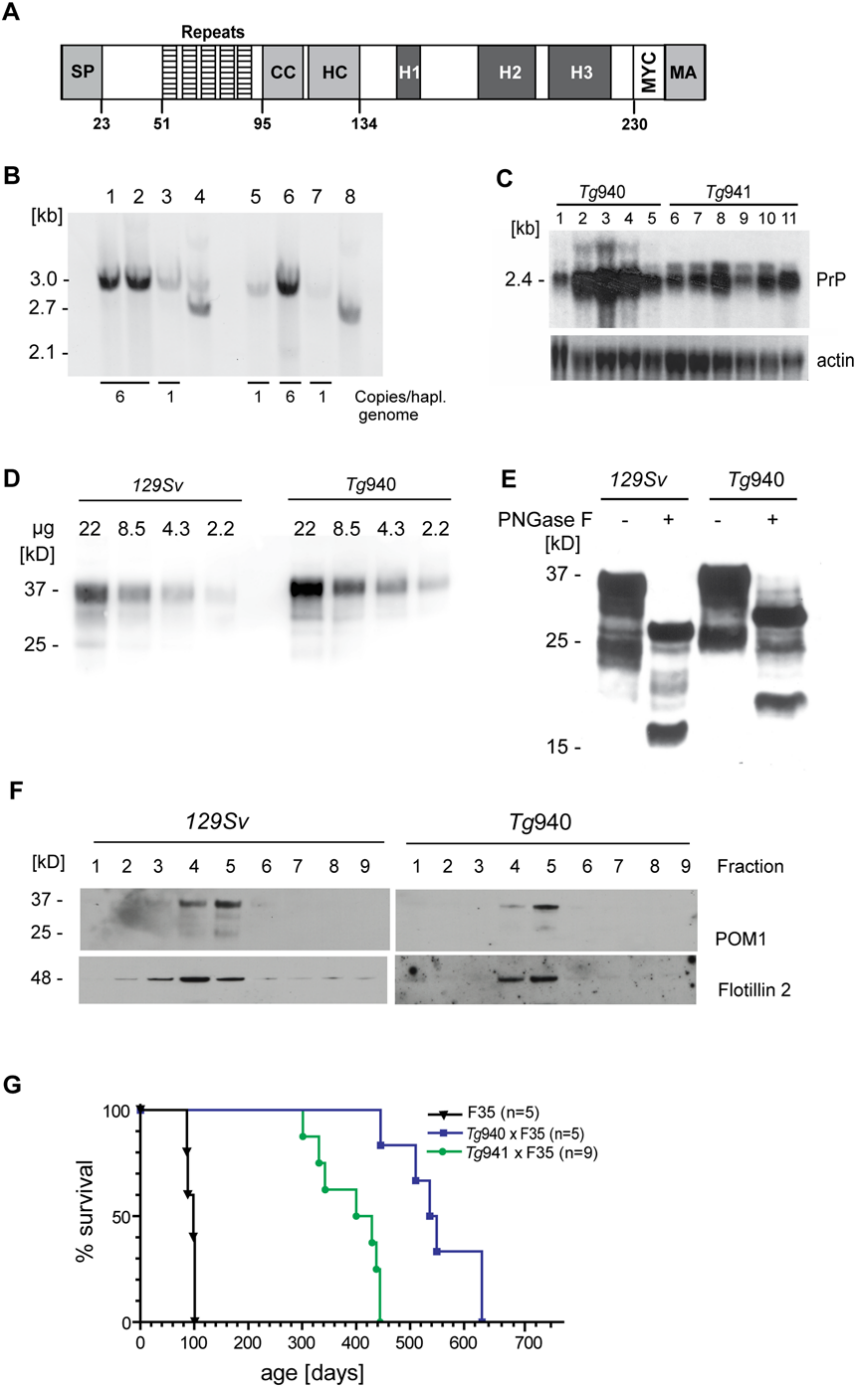
Molecular characterization of the PrP_myc_ transgenic mouse lines *Tg*940 and *Tg*941. (A) Scheme of the PrP_myc_ transgene. SP: secretory signal peptide, cleaved after sorting of the precursor to endoplasmic reticulum; repeats: five repeats of eight amino acids; CC: charge cluster; HC: hydrophobic core; H1, H2, H3: α-helices of the globular carboxy-proximal domain; MYC: human myc epitope tag (EQKLISEEDL); MA: membrane anchor of precursor protein, replaced during maturation with glycosyl phosphatidyl inositol anchor. (B) Southern blot analysis of lines *Tg*940 PrP_myc_ (lanes 1, 2, 6) and *Tg*941 PrP_myc_ (lanes 3, 5, 7). Lane 4: *Tg*941 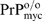 mouse co-expressing N-proximally truncated PrP_ΔF_. Lane 8: PrP_ΔF_ mouse. The bands diagnostic for PrP_myc_ and PrP_ΔF_ were 3039 and 2709 bp, respectively. Numbers of transgenic copies per haploid genome, as determined by quantitation of Southern blot signals against the respective *Prnp* ^o^ genomic band, revealed higher copy numbers in *Tg*940 PrP_myc_ (#6) than in *Tg*941 PrP_myc_ mice (#1). (C) Northern blot analysis of individual *Tg*940 PrP_myc_ and *Tg*941 PrP_myc_ brains using a *Prnp* probe. Mice homozygous for the transgenic allele PrP_myc_ (lanes 2, 3, 4 from *Tg*940 and lanes 8, 11 from *Tg*941) showed higher levels of PrP_myc_ mRNA than hemizygous mice (lanes 1 and 5 from *Tg*940 and lanes 6, 9, 10 from *Tg*941). An actin probe was used as a loading control (lower panel). (D) Similar expression levels of transgenic protein from *Tg*940 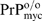, and full-length PrP from *129S2/SvPas* wild-type mice, analyzed by Western blotting of total brain homogenate using anti-PrP antibody POM1. (E) Similar glycosylation pattern of full-length PrP from *129S2/SvPas* wild-type and PrP_myc_ from *Tg*940 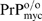 mice. Brain homogenates were subjected to PNGase F treatment as indicated, and analyzed by Western blotting using POM1 antibody to PrP^C^. (F) Detergent-resistant membrane preparations from cerebella of *Tg*940 PrP_myc_ transgenic mice showed PrP_myc_ in lipid rafts. PrP_myc_ was detectable by Western blotting in fractions with 5–30% Optiprep. PrP_myc_ resided in the same fractions as flotillin (48 kDa) confirming its localization in DRMs. (G) A genetic in vivo assay for the function of the PrP_myc_ protein. Survival curves of mice expressing PrP_ΔF_ in absence of full length PrP^C^ and in presence of PrP_myc_ from two transgenic lines. Toxicity of PrP_ΔF_ was counteracted by PrP_myc_, leading to a longer survival and suggesting that PrP_myc_ has retained at least some of the function of PrP^C^. Line PrP_ΔF_, *Tg*940 and *Tg*941 consisted of 5, 5, and 9 individuals, respectively.

We tagged the murine prion protein by introducing a human myc epitope tag (EQKLISEEDL) at its C terminus next to Ser^230^ and amino proximally to the C-terminal signal sequence for the GPI anchor (Fig. 1A). As the minimal myc epitope tag consists of only 10 amino acids, we reasoned that it might not interfere with the geometry and proper folding of PrP^C^, and with its function. The human myc epitope tag was detectable by both monoclonal anti-myc antibodies 9E10 and 4A6 [13]. To guarantee correct GPI linkage of this fusion protein, the sequence comprising Ser^230^ and its four immediately preceding N-proximal amino acids was duplicated after the tag. The resulting fusion molecule was termed PrP_myc_.

Preliminary analyses of PrP_myc_ transfected cells indicate that the biosynthesis, processing, and trafficking of the resulting fusion protein were indistinguishable from those of endogenous PrP^C^ (data not shown).

To generate transgenic mice expressing C-terminally tagged PrP, PrP_myc_ was ligated into the ‘half-genomic’ phgPrP backbone, driven by the endogenous *Prnp* promoter [14]. Pronuclear injections of linearized purified DNA were performed into fertilized oocytes derived from a B6D2F1×B6;129S5-*Prnp* ^o/o^ mating. Four founder mice were identified by PCR analysis using primers TAP 20 (5′- CCG ATG TGA AGA TGA TGG AGC) and myc 22 (5′- CCG TCG ATC GGA TTC AGA TCC) specific for the myc-tag amplicon. The two highest-expressing lines, termed *Tg*(PrP_myc_)940Zbz and *Tg*(PrP_myc_)941Zbz (henceforth *Tg*940 and *Tg*941 for brevity) were chosen for further propagation.

Southern blot analysis revealed that *Tg*940 and *Tg*941 mice harbored 6 copies and 1 copy of the transgene per haploid genome, respectively (Fig. 1B). Northern blot analysis performed on total RNA from brains of PrP_myc_ mice confirmed transcription of transgenic PrP_myc_ (Fig. 1C). Transgenic mice expressing PrP_myc_ did not show any anatomical or behavioral abnormalities, survived in health for >700 days, and did not show any neurohistological changes. We monitored weight and food uptake until adolescence. Transgenic mice had shiny fur indicative of good general health, and reproduced with frequency and litter sizes comparable to wild-type mice (data not shown). We did not recognize any difference in locomotor activity from wild-type mice over a period of >2 years.

To obtain transgenic strains that only expressed PrP_myc_ yet no endogenous PrP, both transgenic founders *Tg*940 and *Tg*941 were crossed twice to *Prnp* ^o/o^ mice. Transgene expression in brain and spleen of these mice was analyzed by Western blotting using anti-PrP antibody POM1 [15], and mouse monoclonal anti-myc antibody 9E10. *Tg*940 mice lacking PrP^C^ (henceforth termed *Tg*940 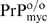) expressed 1.6 fold more of PrP_myc_ protein in brain than wild-type mice (Fig. 1D), but had lower expression levels of the transgene in spleen (about 0.5 fold of *Prnp* ^+/o^ mice, data not shown). Expression of PrP_myc_ in *Tg*941 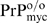 was approximately 0.33 fold in brain and 2-fold in spleen of PrP^C^ expression in *Prnp* ^+/o^ mice (data not shown). *Tg*940 and *Tg*941 exhibited a three-banded pattern very similar to PrP^C^ glycoforms (37–25 kDa) in wild type mice (Fig. 1E).

### PrP_myc_ is localized within detergent resistant membranes (DRMs)

We isolated DRMs from *Tg*940 brain tissue by gradient centrifugation [16]. A series of fifteen individual fractions was carefully removed from the tubes after centrifugation of typical DRM preparations from mouse cerebella of *Tg*940 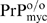, and analyzed by Western blotting. The quality of the preparations was monitored using the control proteins flotillin 2 is known to reside in DRMs [17], [18]. PrP_myc_ was found to reside in the same fractions as these proteins, confirming its localization in these specialized membrane domains (Fig. 1F). Therefore, the subcellular localization of PrP_myc_ was similar to that of endogenous PrP^C^.

### Testing the functionality of PrP_myc_

*Tg*940 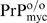 were crossed with the *Tg*F35 line of mice expressing N-proximally truncated PrP, henceforth referred to as PrP_ΔF_. PrP_ΔF_ mice suffer from degeneration of the cerebellar granular layer, leukoencephalopathy, and death at about 100 days of age [19], [20], [21]. This phenotype is dose-dependently counteracted by endogenous or transgenic co-expression of wild-type PrP^C^, presumably because of a competing activity supplied by PrP^C^.

If the tagged protein PrP_myc_ is functional and appropriately localized, it should also rescue PrP_ΔF_ mice from neurodegeneration. Indeed, *Tg*940 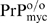 expressing PrP_ΔF_ survived for 551±73 days (n = 5; Fig. 1G) and maintained a normal weight throughout their lifetime. Mice were examined twice per week for neurological symptoms and scored as described [19], yet did not show clinical signs of CNS disease at any time. Furthermore, they did not develop histopathological changes in brain or other organs (data not shown), suggesting that PrP_myc_ is functional *in vivo*. Age and sex-matched PrP_ΔF_ siblings died between 12 and 14 weeks of age (mean survival: 95±7 days, n = 5; Fig. 1G).

In contrast, double-transgenic mice of the lower expressing line (*Tg*941) were not completely rescued and began to show first signs of illness around day 280. Some animals had to be sacrificed at the age of 12 months due to hind leg paresis (mean survival 391±57 days, n = 9; Fig. 1G). As *Tg*941 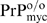 mice express about one-third of the PrP_myc_ found in brains of *Tg*940 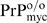 mice, this indicates that the action of PrP_myc_, like that of PrP^C^, is dose-dependent.

### Neuropathology in inoculated 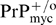 mice

**Figure 2:**
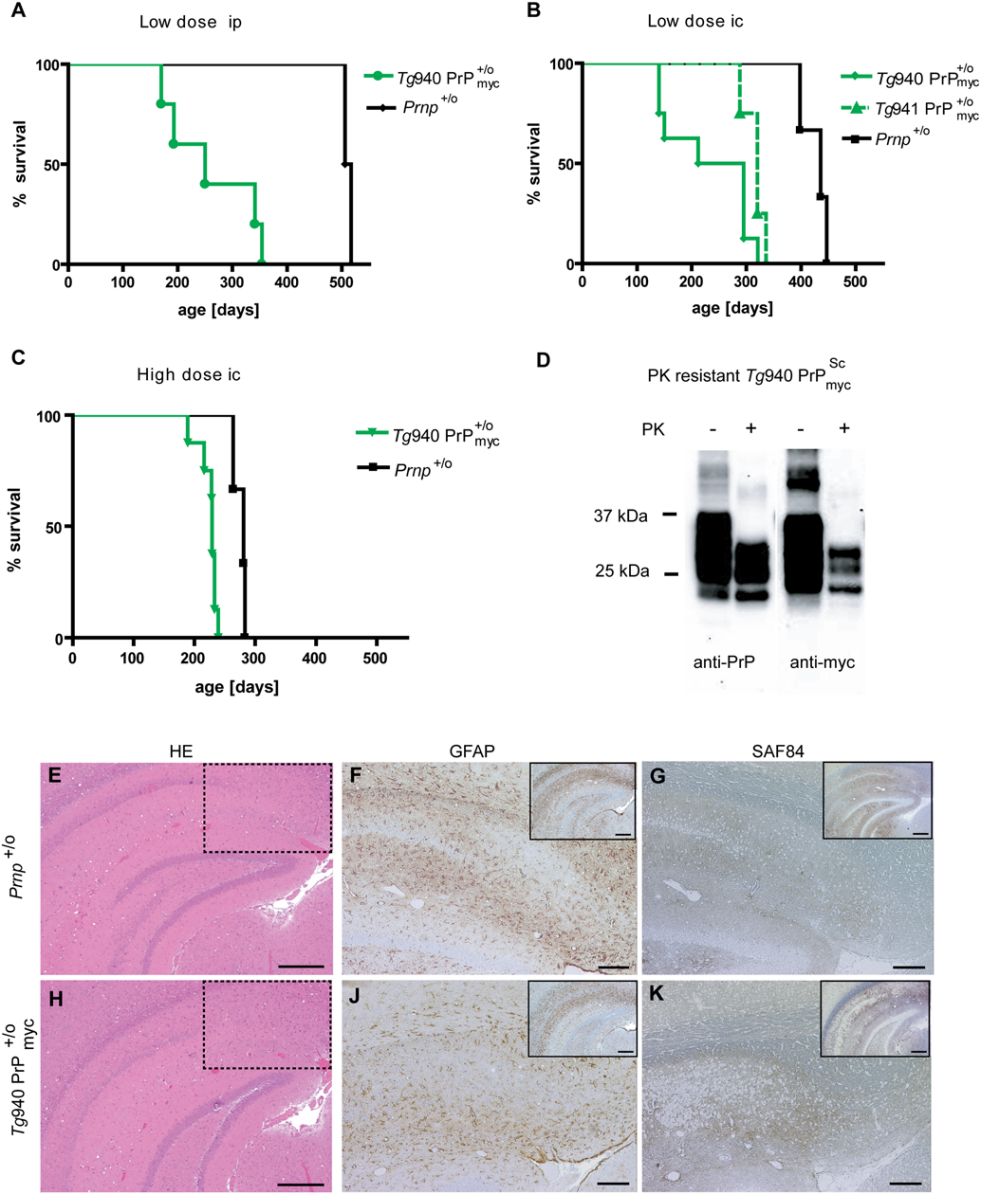
Survival and neuropathology of 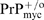 mice after prion inoculation. (A) Survival curves of *Tg*940 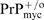 mice and *Prnp* ^+/o^ mice low dose ip inoculated with RML5 prions. Groups *Tg*940 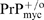 ip *Prnp* ^+/o^ ip consisted of 5 and 2 individuals, respectively. (B) Survival curves of *Tg*940 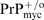, *Tg*941 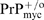, and *Prnp* ^+/o^ mice inoculated low dose ic with RML5 prions. Group *Tg*940 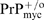 comprises 6, group *Tg*941 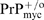 4, and *Prnp* ^+/o^ 3 individuals, respectively. (C) Survival curves of *Tg*940 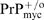 mice and *Prnp* ^+/o^ mice high dose ic inoculated with RML5 prions. Line *Tg*940 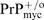 comprises of 8 and line *Prnp* ^+/o^ of 6 individuals, respectively. (D) PrP_myc_ was converted into myc-tagged proteinase K-resistant 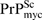 in presence of a wild-type PrP allele. Western blot analysis using brain homogenate from an inoculated, terminally sick 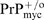 mouse. Antibodies POM1 to PrP and 4A6 to myc were used for detection. Samples were treated with PK as indicated, revealing the presence of protease resistant PrP_myc_ in the brain of inoculated *Tg*940 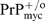 mice. (E–G) Similar neuropathological changes in hippocampus of a RML inoculated *Prnp* ^+/o^ mouse and (H–K) a RML-inoculated *Tg*940 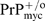 mouse. (E, H) Hematoxylin-eosin stains showing vacuolar degeneration and nerve cell loss. The dashed lines indicate the magnified area shown in F,G,J and K. Scale bar = 500 µm. (F, J) GFAP immunohistochemistry for the detection of reactive astrocytes and (G, K) mAb SAF84 for PrP aggregates. Scale bar = 200 µm. The small inserts represent the low magnification pictures of the GFAP and SAF84 stained sections consecutive to E and H. Scale bar = 500 µm.

To assess whether PrP_myc_ can be converted into myc-tagged protease-resistant 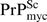, 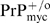 and 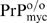 mice from lines *Tg*940 and *Tg*941 were inoculated with mouse-adapted sheep prions (RML strain, passage 5). After low dose intraperitoneal (ip) inoculation with 10^3^ IU or intracerebral (ic) inoculation with 300 IU of RML5 brain homogenate, *Tg*940 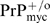 mice showed signs of CNS dysfunction at 250±92 (n = 5/5) and 236±76 (n = 6/6) days post inoculation (dpi), respectively (Fig. 2A and B). Mice expressing less PrP_myc_ in brain (*Tg*941) developed signs of CNS dysfunction and terminal scrapie disease more slowly, at 316±20 (n = 4/4) days after low-dose intracerebral inoculation (Fig. 2B and Table S1).

Brain homogenates prepared from terminally sick *Tg*940 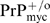 mice were inoculated ic into *tga*20 mice overexpressing PrP^C^ [14] to test for infectivity in an in-vivo mouse assay. All of the *tga*20 mice developed neurological signs of terminal scrapie at around 80 dpi (Table S1). Prion infection was confirmed by immunochemical and histopathological analysis in all terminally sick mice. 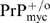 mice developed neurological dysfunction and terminal disease significantly earlier than *Prnp* ^+/o^ mice: the mean incubation time was 276±9 days for *Prnp* ^+/o^ (n = 6) and 226±13 days for *Tg*940 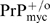 mice (n = 8) after high dose ic inoculation (Fig. 2C and Table S1). Therefore, PrP_myc_ contributes to, rather than interfering with, prion pathogenesis in *Prnp* ^+/o^ mice.

In all terminally sick 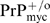 mice tested we detected proteinase K (PK) resistant material in brain and spleen after ic or ip inoculation with RML prions. To distinguish between wild-type PrP^Sc^ and 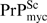 we stained Western blots of brain homogenates with an anti-myc antibody (Fig. 2D). PK-resistant 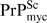 was clearly detectable under these conditions, indicating that PrP_myc_ itself is convertible, and suggesting that this phenomenon contributed to the shortened incubation periods in 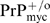 mice. Comparison of immunohistochemically stained brain sections of terminal *Prnp* ^+/o^ and *Tg*940 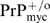 mice did not reveal any striking differences in the extent and topography of reactive astrocytic gliosis, vacuolar degeneration and PrP aggregates (Fig. 2E–K).

### Neuropathology in inoculated 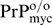 mice

**Figure 3:**
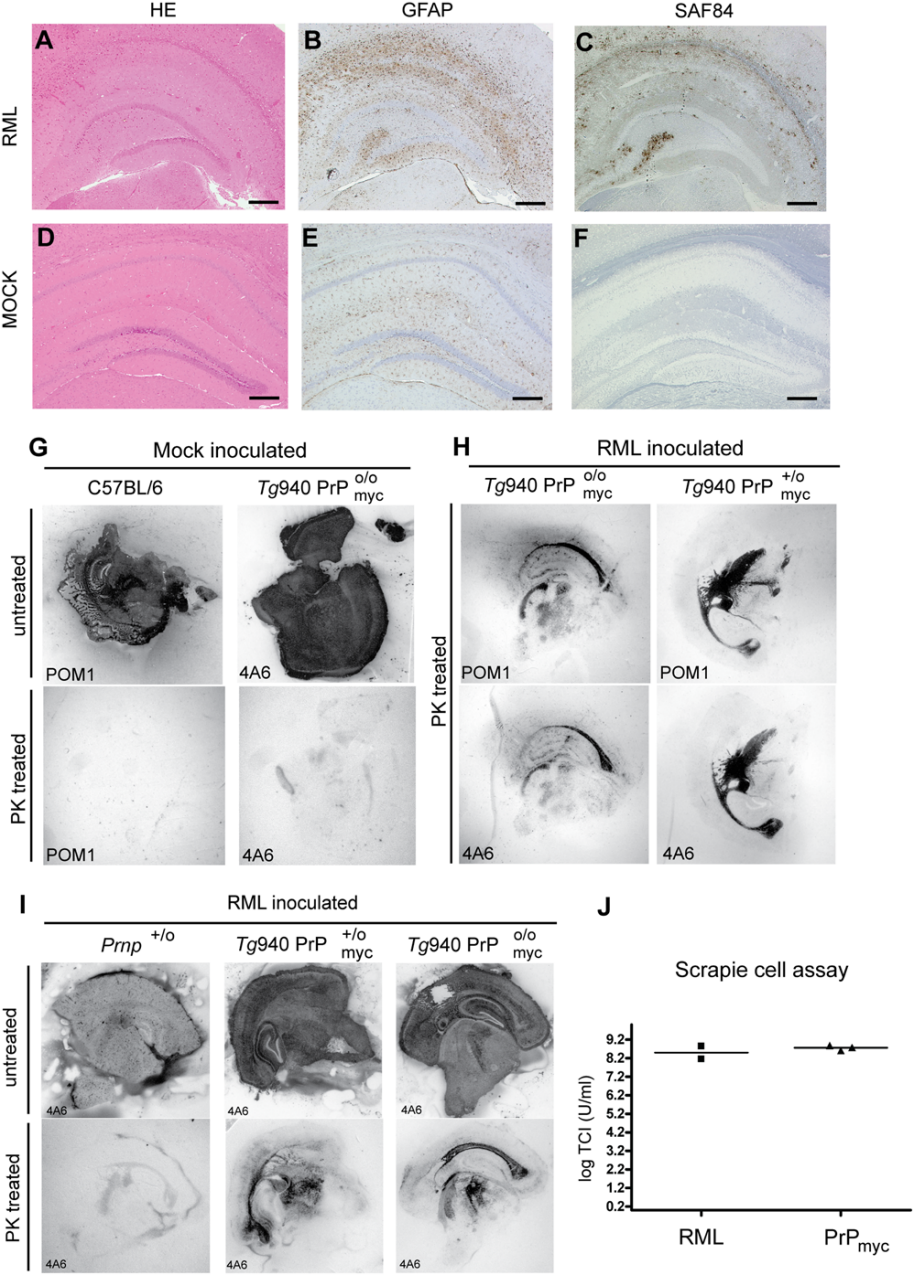
Neuropathology of *Tg*940 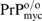 mice after prion inoculation. (A–C) Extensive astrogliosis and PrP aggregation in hippocampus of an RML inoculated *Tg*940 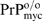 mouse compared to (D–F) Mock inoculated *Tg*940 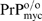 mouse: (A, D) Hematoxylin-eosin stains visualizing vacuolar degeneration and nerve cell loss, (B, E) GFAP immunohistochemistry indicating reactive astrocytic gliosis, and (C, F) mAb SAF84 showing PrP_myc_ aggregates. Scale bar = 500 µm. (G–I) Conversion of PrP_myc_ into myc-tagged PK-resistant 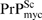 in mice lacking both wild-type *Prnp* alleles. Histoblot analysis of coronal slices from brains of mock and prion-inoculated mice blotted onto nitrocellulose membranes. (G) Mock-inoculated C57BL/6 and *Tg*940 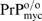 mice. Brain homogenates were incubated with POM1 and 4A6 before or after PK treatment, and showed no PK-resistant PrP. (H) Prion-inoculated *Tg*940 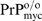 and *Tg*940 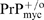 mice, treated with PK and incubated with POM1 and 4A6, showed PK-resistant material in brain. (I) Prion-inoculated *Prnp* ^+/o^, *Tg*940 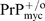 and *Tg*940 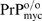 mice treated with PK and untreated were stained with 4A6 anti-myc antibody and show protease-resistant PrP_myc_ in the brain. A terminally sick *Prnp* ^+/o^ mouse was used to control for nonspecific 4A6 signals. (J) SCEPA of brain homogenates of 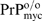 and wild-type mouse. Three independent biological replicas of 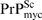 and 2 independent biological replicas for RML were analyzed in tenfold dilution steps using 6–12 PK1-containing replica wells for each dilution. Data points indicate the number of infectious tissue culture units per ml of brain homogenates.

To investigate whether PrP_myc_ can be converted into myc-tagged PK-resistant 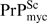 even in the absence of a wild-type PrP allele, we inoculated 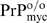 mice with RML prions. No PrP^Sc^ was detected in brain and spleen at 50 to 100 days after ic or ip inoculation, yet 8 of 34 (23%) 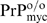 mice eventually developed a progressive neurological syndrome clinically indistinguishable from scrapie after RML inoculation (Table S2). Brain homogenate from these sick mice was then used to inoculate a second generation of *Tg*940 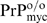 mice. Western blot analysis of brain homogenate from these second-passage ic-inoculated *Tg*940 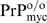 mice revealed PK-resistant PrP; these mice had clinical signs of scrapie and developed vacuolation in the neuropil, intense astrogliosis, and abundant PrP aggregates (Fig. 3A–C). For control, *Tg*940 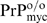 mice were inoculated with non-infectious brain homogenate. These mice showed no evidence of vacuolar degeneration or nerve cell loss, and only mild astrogliosis when aged (Fig. 3D–F).

As an additional method to distinguish between PrP^Sc^ derived from wild-type PrP and PrP_myc_ we performed histoblot analysis of cryosections of terminal *Tg*940 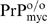 mice and *Tg*940 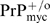 mice (Fig. 3G–I). Using anti-PrP (POM1) and anti-myc (4A6) antibodies, we could specifically detect PK-resistant PrP in terminal C57BL/6 mice, *Tg*940 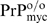 and *Tg*940 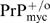 mice. This technique allowed us to map the distribution of PrP^Sc^ in different transgenic mice.

We then investigated whether PrP_myc_ infectivity would increase upon serial transmission, as frequently observed in strain adaptation [22]. Brain homogenate derived from RML-inoculated *Tg*940 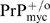 mice was passaged into *Tg*940 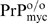 mice which all got sick after 590±56 days (n = 3) (Table S3). One of these second-passage mice was used as a source for a third passage into 5 *Tg*940 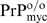 mice. All of them show similar neurological signs as in the second passage, but with a shorter incubation period of 367±38 (n = 5), which is suggestive of strain adaptation (Table S3).

We then tested whether deposition of PrP^Sc^ accompanies prion replication, defined as increase in prion infectivity. Samples from *Tg*940 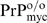 mice after the second passage were used to infect the PK1 subclone of N2a neuroblastoma cells in the Scrapie cell assay in endpoint format (SCEPA [23]). As shown in the Fig. 3 J the titer for the 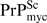 is the same as the standard RML.

### Identification of PrP_myc_ -containing protein complexes

**Figure 4:**
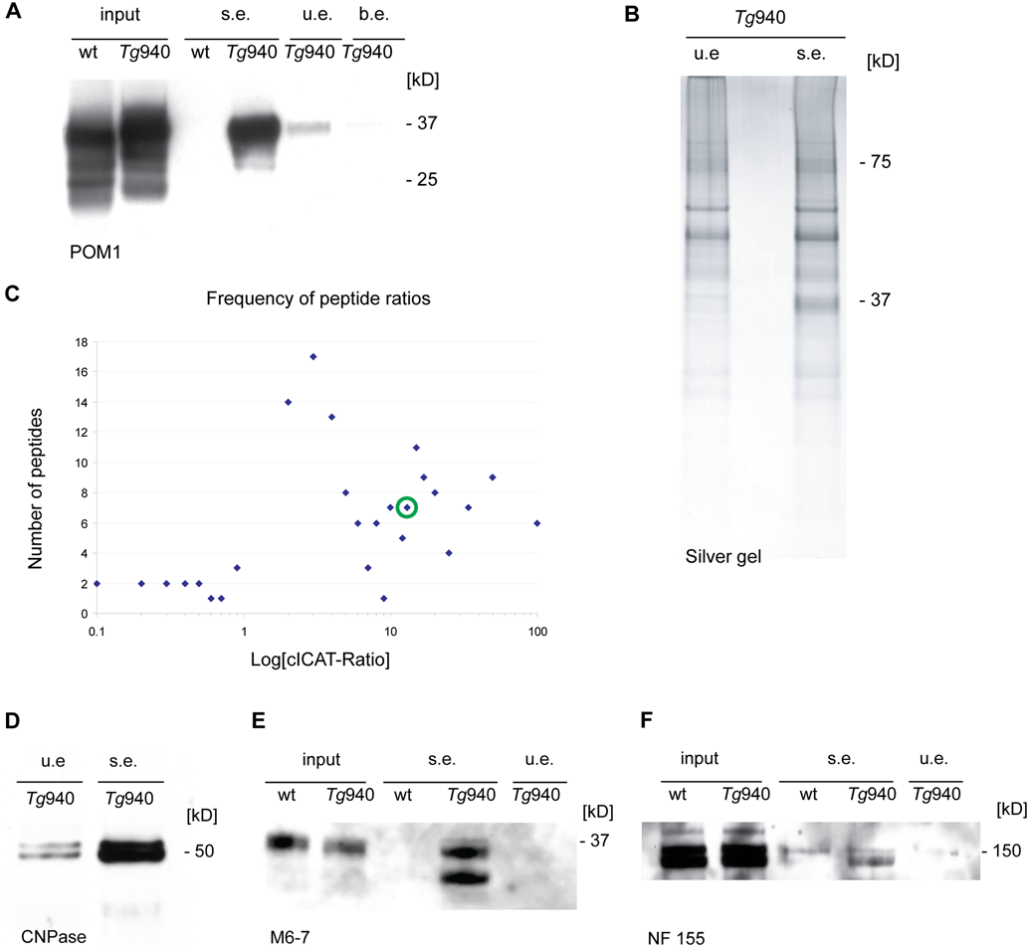
Isolation of PrP_myc_ and 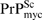 from *Tg*940 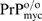 and *Tg*940 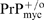 brains. (A) Western blot analysis of the material used for IP of PrP^C^. Equal amounts of brain homogenates from wild-type *129S2/SvPas* and *Tg*940 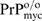 mice were used for immunoprecipitations. Specifically eluted PrP_myc_ protein was detected with anti-PrP antibodies as well as 4A6 anti-myc antibody (data not shown). No signal for PrP in the specific elution (s.e.) from the precipitation in *129S2/SvPas* brain homogenate, and only weak signals from the elutions with unspecific peptide (u.e.), or with PBS only (b.e.). (B) Silver stain of the material immunocaptured with anti-myc antibodies from total brain homogenate of *Tg*940 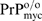 and eluted with *myc* and *cym* peptide. The gel was subsequently used for GeLC-MS/MS experiments. (C) Blot of peptide pair frequency against XPRESS-Ratios on a logarithmic scale. Values of ratios where one of the two labeled peptide was not detected (1∶0 or 0∶1) were excluded from the dataset. The ratio of the cystein-containing peptide pair of PrP heavy/light is indicated by the green circle. (D–F) Western blot analysis of those protein candidates listed in Table 1 for which specific antibodies were available. (D) Western blot analysis of the specific and scrambled-peptide IP elution using anti-CNPase antibody. (E–F) Western blot analyses of IP input material from wild-type *129S2/SvPas* and *Tg*940 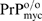 mice and specific and unspecific peptide elution using anti-M6 (M6-7) and anti-Neurofascin (NF155) antibodies.

Crude brain homogenates from *Tg*940 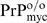 mice were subjected to immunoprecipitation (IP) experiments with paramagnetic microbeads coupled to mouse monoclonal anti-myc antibody (4A6, Upstate, USA). Release of myc-containing protein complexes from beads was carried out by exposing the beads to an excess of the synthetic epitope-mimicking myc peptide described above. Control experiments were carried out to verify the specificity of the eluted proteins, and included (1) incubation of beads with *129S2/SvPas* wild-type brains followed by elution with the myc peptide, as well as (2) incubation of beads with *Tg*940 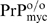 homogenate followed by elution with a scrambled version of the myc peptide. In the eluates from 4A6-coupled beads incubated with *129S2/SvPas* wild-type brain homogenates, PrP^C^ was not detected, whereas only traces of PrP^C^ were detected in the scrambled-peptide eluate from IPs of *Tg*940 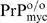 brain homogenates (Fig. 4A).

Inspection of silver-stained gels revealed more protein bands in the specific than in the unspecific elution fraction (Fig. 4B), in particular the PrP_myc_ band exclusively present in the myc-specific eluates from *Tg*940 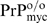 brain homogenates. The corresponding lanes were cut into slices, proteins were extracted, and tryptic peptides were identified by liquid chromatography followed by tandem mass spectrometry (LC-MS/MS). As a further quality control, we verified that the identified proteins originated from the gel area corresponding to their predicted molecular size. Table S4 lists those proteins that were coprecipitated with PrP_myc_ from transgenic brains, yet were not detected in material immunoprecipitated from wild-type brains and unspecific elution under the same conditions. While 442 individual proteins were detected in both the specific and the nonspecific eluates, and 277 proteins were uniquely present in the nonspecific eluate, 96 proteins were present in the specific eluate but absent from the nonspecific eluate.

We then sought to determine the relative abundance of PrP and the interacting proteins in the specific and unspecific peptide elution fractions by using cleavable isotope-coded affinity tags (cICAT) as a quantitative mass spectrometric technique. In the classical cICAT approach the two labeled fractions contain the same amount of protein. Since this is not the case for the specific and unspecific IP elution fractions, we could only determine the relative ratio of PrP between the specific and the unspecific elution fractions.

The two elution fractions derived from immunoprecipitations of PrP_myc_ and wild-type brains were labeled with the “heavy” (cICAT-13C9) and “light” (cICAT-12C9) cICAT tags, mixed, and mass/charge (m/z) elution profiles were determined by mass spectrometry. Sequest [24], PeptideProphet [25] and XPRESS were used to identify the proteins and to access the cICAT ratios (Fig. 4C, Table S5). Of the 157 peptide pairs that could be assigned to a heavy/light ratio between 0.1 and 100, seven proteins were found to have a comparable ratio to PrP and, at the same time, were identified as specific proteins by the gel-based approach (Table 1). Any ratios below 1 are indicative of proteins more abundant in the scrambled elution than in the myc-specific elution. Proteins displaying a similar abundance in both samples would yield a ratio of 1, which most probably indicates nonspecific binding to and elution from the beads. The ratio for PrP was about 14, and the proteins listed in Table 1 represent values between 4 and 15.

**Table 1:** Proteins found by GeLC-MS/MS and cICAT experiments.

UniProt accession number	Protein name and cICAT peptide sequences	Gene	Function/localization (ExPASy)
P16330	2′,3′-cyclic-nucleotide 3′-phosphodiesterase, (RPPGVLHCTTK, LDEDLAGYCRR)	*Cnp*	Associated with membrane structures of brain white matter
Q62059	Chondroitin sulfate proteoglycan core protein 2 (Large fibroblast proteoglycan, PG-M), (YHCKDGFIQR, YQCDEGFSQHR)	*Cspg2*	May play a role in intercellular signaling and in connecting cells with the extracellular matrix. May take part in the regulation of cell motility, growth and differentiation.
P35802	Neuronal membrane glycoprotein M6-a, (KICTASENFLR)	*Gpm6a*	Multi-pass membrane protein. Enriched in the granule cell layer of the cerebellum but not in the molecular layer or white matter. Belongs to the myelin proteolipid protein family.
Q810U3	Neurofascin, (RGTTVQLECR)	*Nfasc*	Single-pass type I membrane protein. Cell adhesion, ankyrin-binding protein which may be involved in neurite extension, axonal guidance, synaptogenesis, myelination and neuron-glial cell interactions
P04925	Major prion protein, (VVEQMCVTQYQK, VVEQMCVTQYQKESQAYYDGR)	*Prnp*	Cellular prion protein.
Q80U89	MKIAA0034 protein (clathrin, heavy polypeptide (HC)), (YIQAACKTGQIKEVER, IHGCEEPATHNALAK)	*Cltc*	Coated pits.
Q01853	Valosin containing protein, transitional endoplasmic reticulum ATPase, (FGMTPSKGVLFYGPPGCGRK)	*Vcp*	Necessary for the fragmentation of Golgi stacks during mitosis and for their reassembly after mitosis

We then sought to confirm the results of mass spectrometric analyses by immunochemical analyses of selected proteins. Indeed, the identity of PrP, 2′,3′-cyclic nucleotide 3′-phophodiesterase, M6-a and Neurofascin was unambiguously confirmed by Western blot analysis. Fig. 4D shows the characteristic double band of CNPase after myc-peptide elution and a low-intensity band for the scrambled-peptide elution. Western blot analysis with antibodies to Neurofascin 155 and M6a revealed specific bands for the specific-peptide elution but in none of the negative controls (Fig. 4E–F). The signal for M6a from the specific elution shows two strong bands most probably originating from alternative splicing. For both Neurofascin and M6a, the protein expression level in wt and *Tg*940 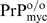 brain were approximately the same as illustrated in Fig. 4E–F.

## Discussion

Our understanding of the function of PrP^C^ and its conversion into PrP^Sc^ continues to be sketchy. Genetic experiments have helped defining the domains of PrP^C^ necessary for prion propagation [21] and, with some limitations, for PrP^C^ function [19], [26], [27], [28], yet have failed to identify any further proteins that may be required for this process. However, progress in this field may crucially benefit from enumerating and/or manipulating the PrP-interacting proteome. Towards the latter goals, we have studied the biogenesis, localization *in vitro* and *in vivo* of a C-terminally myc-tagged version of PrP^C^ (PrP_myc_). Since the physiological function of PrP^C^ is unknown, we used a well-established approach of reverse genetics [14] to assay the biological activity of PrP_myc_. This approach is so far the most proximal surrogate to study the function of PrP. We found PrP_myc_ to be fully functional and substitute dosage-dependently for endogenous PrP in rescuing the neurodegenerative phenotype induced by PrP_ΔF_.

Conversion of cellular prion protein PrP^C^ into the disease-causing isoform PrP^Sc^ is the central pathogenic process in prion diseases [29]. Therefore, any claim of the biological authenticity of a modified PrP protein should be substantiated by its ability to sustain prion replication. We approached this important question in a variety of paradigms. Whereas direct intracerebral inoculation of 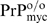 transgenic mice with prions rarely induced scrapie, we found that in the presence of a wild-type *Prnp* allele PrP_myc_ is converted into a PK-resistant isoform (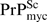). The disease of prion-infected 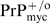 mice was transmissible by ic inoculation of brain homogenates to wild-type mice and also, importantly, to 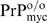 mice. Since it is known, that PrP^Sc^ levels do not necessarily correlate with infectivity titers, we decide to evaluate the infectivity titers by SCEPA and compare to RML, and also in that paradigm PrPmyc behave as normal RML. The latter finding establishes beyond any doubt that PrP_myc_ supports prion replication and scrapie pathogenesis.

In many paradigms, expression of heterologous PrP molecules which differ from the endogenous PrP by as little as one amino acid can profoundly interfere with the overall accumulation of PrP^Sc^ [30], [31], suggesting that precise homotypic interactions between PrP molecules are important for PrP^Sc^ accumulation [31], [32]. However, when inoculated with the same dose of prions, 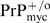 mice developed disease faster than *Prnp* ^+/o^ mice, implying that PrP_myc_ cooperates, rather than interfering, with PrP^C^ in disease pathogenesis. This was unexpected in view of the many instances of interference that have documented to occur even between naturally occurring PrP alleles [12]. If one accepts that interference is brought about by disturbances of the replicative interface of prions, one might speculate that the carboxy terminus of PrP^C^ does not participate to such an interface.

The latter conclusion, however, is tempered by another observation. When 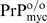 mice were inoculated with RML prions, only few animals developed clinical signs of scrapie. This suggests that the C-terminally modified prion protein presents a “prion transmission barrier” to mouse-adapted sheep prions, analogously to the species barriers seen in many natural and experimental prion diseases [33]. The similarities between the amino acid sequence of donor PrP^Sc^ and recipient PrP^C^ play a crucial role in the species barrier [34], [35], but the structural understanding of these constraints is still very sketchy. In the PrP_myc_ transgenic model, the species barrier exists if wild-type prions are transmitted into 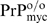 animals, but can be overcome if brain homogenates from terminally sick 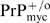 mice containing 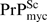 is passaged into 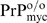 transgenic mice.

The successful production of myc-tagged, self-propagating and disease-causing prions paves the way to many studies *in vitro* and *in vivo* by taking advantage of the high-affinity reagents available to the myc epitope. For example, the myc-tagged prion inoculum may allow for investigating the fate of inoculated prions *in vivo*, since PrP_myc_ can be detected and traced by tag-specific antibodies which do not recognize endogenous PrP. In the present study, we provide evidence that PrP_myc_ is useful for probing the PrP^C^-associated proteome. We have established a novel method for the specific elution of multiprotein complexes containing PrP_myc_. We have exploited this method for identifying several candidate proteins which appear to interact with PrP^C^ *in vivo*. The specificity of these interactions was validated by comparison to wild-type brain eluates and elution with a scrambled peptide. Some of the PrP-interacting proteins describe before and summarized in recent reviews [36], [37], including for instance Tubulin, Hsp60 and Laminin, were detected in the specific as well as unspecific elution fraction of our approach and therefore not included into the list of possible candidates.

We utilized a quantitative MS technique, isotope-coded affinity tagging (ICAT), to determine the relative abundance of PrP and other proteins in the various samples, so to identify proteins that might exist in an equimolar complex with PrP^C^. Such PrP_myc_-interacting proteins would display an ICAT ratio of specific/unspecific signals similar to that of PrP^C^. Based on this mass spectrometric approach, we found a small number of protein candidates equimolarly associated with PrP_myc_ in native brain homogenates.

There are some caveats to the equimolarity filter described above. Supramolecular complexes encompassing PrP^C^ may contain superstoichiometric amounts of accompanying molecules, in which case the ICAT ratios may be skewed. Conversely, if PrP^C^ exists in a free form as well as in a complex, or in several different complexes, the partner proteins may appear to be substoichiometric in an immunoprecipitate. Therefore, even if the seven proteins identified here represent promising candidates, the remaining hits detailed in Table S4 should not be dismissed because of their non-equimolar ICAT ratios.

Two of the latter seven proteins (Q80U89 clathrin linked; Q01853 translational ER ATPase) are not well-characterized and no antibodies to them appear to be available. Chondroitin sulfate proteoglycan core protein was described to strongly inhibit neurite outgrowth of central and peripheral neurons [38]. It was also reported that neurite outgrowth is modulated – at least in culture models – by interactions between PrP^C^, NCAM and STI-1, which can lead to activation of intracellular signalling pathway [39].

Several PrP_myc_ interactors belong to the families of neuronal glycoproteins and myelin-associated proteins. These include the neuronal membrane glycoprotein M6-a, Neurofascin, and 2′,3′-cyclic nucleotide 3′-phophodiesterase (CNP). P0 glycoprotein of compact PNS myelin, myelin-associated glycoprotein (MAG), and others have well-defined roles in the formation, maintenance and degeneration of myelin sheaths [40]. Myelin proteins also appear to mediate signals between the myelin-forming cell and the axon [41]. Current research suggests that CNP is required for maintenance of axon-glial interactions at the nodes of Ranvier in the CNS [42]. The interaction between PrP and CNP may underlie the myelin damage observed in old *Prnp* ^o/o^ mice [43] and in various transgenic PrP deletion mutants age [19], [20], [21]. In support of this hypothesis, recent studies suggest that myelin integrity may be maintained by a constitutively active neurotrophic protein complex involving PrP^C^ [19].

A possible functional relation between neurofascin and PrP^C^ is particularly intriguing in view of the lethal phenotype of transgenic mice expressing PrP deletion mutants, which display extensive central and peripheral myelin degeneration [19]. Neurofascin 186 (NF186) is expressed prenatally on dorsal root ganglia neurons and it may modulate their adhesive interactions with Schwann cells, which express NF155 postnatally and require it for development of axon–glial paranodal junctions. The major isoform of NF186 inhibits cell adhesion, and this activity may be important in formation of the node of Ranvier [44].

Another enticing candidate for functionally relevant interactions is M6-a, a membrane glycoprotein involved in neuronal differentiation as part of a Ca^2+^ channel [45]. The lack of the cellular prion protein was shown to affect Ca^2+^ homeostasis in neurons [46], and therefore it is thinkable that PrP^C^ and M6-a are involved in a complex possessing an ion channel-like function.

In addition to identifying the interactors described above, the tools introduced here may allow for studying supramolecular complexes containing the disease-associated prion protein PrP^Sc^. The biophysical properties and aggregational state of PrP^Sc^ are vastly different from those of PrP^C^, and there is reason to hypothesize that the PrP^Sc^ interactome will only partially overlap with that of PrP^C^. Since most prion strains are both neurotropic and lymphotropic [47], [48], and inflammatory conditions specify the tropism of prions [49], [50], the interactome of PrP^C^ and PrP^Sc^ in lymphoid organs will also be of interest. The inoculation of wild-type animals with myc-tagged prions may help elucidating the initial events that occur during infection of an animal with prions. Finally, the successful conversion of PrP_myc_ into a protease-resistant moiety may allow for the purification of native PrP^Sc^-containing complexes using the techniques described above for PrP^C^. The latter studies may lead to the identification of the elusive chaperones involved in prion propagation, strain barriers and strain adaptation, as well as the crossing of prion species barriers.

## Materials and Methods

### Generation of myc-tagged PrP^C^

PCRs were performed in 50 µl volumes containing 10 ng of template DNA phgPrP [14], 200 µM of each dNTP, 20 pmol of each primer (*Pm*l: 5′-TTT TTT TTC ACG TGT GGA TGC TCT AGC TAT CCC AGG TGG GA-3′, *Cla*I: 5′-TTT TTT TTA TCG ATC GAC GGC AGA AGA TCG AGC AGC ACC GTG CTT TTC TCC TCC CCT CCT GTC ATC-3′, Xma: 5′-TTT TTT TTC CCG GGC AGG GAA GCC CTG GAG GCA ACC GTT-3′, *Cla*I: 5′-TTT TTT TTA TCG ATC TTC TCC CGT CGT AAT AGG CCT GGG ACT C-3′), 1 µl of ”Advantage II” polymerase (Clontech), 10 µl of 10× reaction buffer supplied by the manufacturer (Clontech). Reaction mixtures were kept at 94°C for 5 min in a thermocycler to inactivate the blocking antibody, ^,^and cycled 30 times. The two PCR products of PrP cDNA were cleaved with *Cla*I and ligated into the pGEM-T easy vector system (TA cloning vector, Promega), generating plasmid pGEM-PrP(*Xma*-*Pml*); *Cla*I. The final insert of pGEM-PrP(*Xma*-*Pml*); *Cla*I consists of a mutated PrP cDNA fragment extending from the *Xma*I restriction site of the PrP ORF to the *Pml*i restriction site located 3′ of the PrP coding region. The myc tag was inserted into the unique *Cla*I site of pGEM-PrP(*Xma*-*Pml*); *Cla*I. Two synthetic 5′-phosphorylated oligonucleotides were annealed (myc-fwd: 5′-CGG AAC AAA AAC TCA TCT CAG AAG AGG ATC TGA ATC; myc-rev: 5′-CGG ATT CAG ATC CTC TTC TGA GAT GAG TTT TTG TTC) to produce a double-stranded DNA with 5′-protruding, *Cla*I compatible ends (myc-tag). The myc-fwd oligonucleotide sequence encodes the human myc epitope, EQKLISEEDL. The myc-tag was ligated into *Cla*I digested pGEM-PrP(*Xma*-*Pml*); *Cla*I generating pGEM-PrP-myc(*Xma*-*Pml*); *Cla*I. Finally, the *Xma*I-*Pml*I fragment of phgPrP [14] was replaced by the *Xma*I-*Pml*I fragment of pGEM-PrP-myc(*Xma*-*Pml*); *Cla*I yielding plasmids phgPrP-myc and the construct was verified by sequencing.

### Generation and characterization of transgenic mice

The phgPrP-myc plasmid, driven by the endogenous *Prnp* promoter in the context of the PrP “half-genomic” construct (phgPrP) [14], was digested with *Not*I and *Sal*I to remove its prokaryotic backbone. Pronuclear injections were performed into fertilized oocytes derived from a B6D2F1×B6;129S5-*Prnp* ^o/o^ mating.To obtain PrP_myc_ transgenic animals on a *Prnp* ^o/o^ knockout background, the founders were backcrossed to homozygous B6;129S5-*Prnp* ^o/o^ mice. To differentiate PrP_myc_ transgenic littermates with *Prnp* ^+/o^ and *Prnp* ^o/o^ genotype the presence of the endogenous *Prnp* ^+^ allele was tested by PCR analysis using primers *Prnp* intron 2 (5′-ATA CTG GGC ACT GAT ACC TTG TTC CTC AT) and P10rev (reverse complementary of P10 5′-GCT GGG CTT GTT CCA CTG ATT ATG GGT AC) amplifying a 352 bp product for the *Prnp* wild-type allele but no PCR product for the *Prnp* ^o^ allele.

For Northern blot analyses, RNA was extracted using Trizol (Invitrogen). A randomly ^32^P-labeled (Rediprime II Random Prime Labelling System, Amersham Biosciences) restriction fragment encompassing all of exons 1 and 2, all of the ORF and a part of exon 3 (*Xba*I-fragment) was used as a PrP probe. This probe hybridizes with all wild-type and tagged PrP mRNAs as well as the “readthrough” RNA from the disrupted *Prnp* locus [51].

Southern blot analyses were performed using a 640 bp DNA probe synthesized by incorporation of digoxigenin-11-dUTP (Roche, Switzerland) during PCR using PrP-specific primers and hybridization was performed following established protocols [52]. For the actin control the Northern blot was probed with an in-house generated mouse beta-actin probe cloned from full-length cDNA.

### Rescue of Shmerling's disease


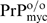
 mice were crossed with PrP_ΔF_ [19], [21] mice to obtain double transgenic animals with *Prnp* ^o/o^ genotype needed for the experiment described in Fig. 1. Animals were examined twice each week for symptoms of cerebellar dysfunction, including ataxia [53], tremor, weight loss, rough hair coat, and kyphosis. Scoring of neurological signs was performed according to a four-degree clinical score system [19] and mice were euthanized within 3 days of reaching a score of 3.5.

### Western blot analyses

Homogenates of noninfectious brain and spleen (10% w/v) were prepared in sterile PBS/0.5% Nonidet P-40 and protease inhibitors (Complete; Roche, Switzerland) by repeated extrusion through syringe needles of successively smaller size. Homogenates of infectious brains were generated using a rhybolyzer in a biosafety level 3 laboratory. After centrifugation for 10 min at 2'400 rpm at 4°C, supernatant was loaded onto 12% SDS-polyacrylamide gels. Proteins were transferred to nitrocellulose membranes (Schleicher & Schuell, Germany) by wet blotting, and first exposed to mouse monoclonal anti-PrP antibody POM-1 [15], 1∶10'000 or mouse monoclonal anti-myc antibody 4A6 (1∶1000, Upstate, USA ), then to peroxidase-labeled rabbit anti-mouse antiserum (1∶10000; Zymed, CA, USA) and developed using the ECL detection system (Pierce, USA). Antibody incubations were performed in 1% Top Block (FLUKA, Switzerland) in PBS-Tween for 1 hour at room temperature or overnight at 4°C. The same protocol was applied to generate Western blots shown in Fig. 4 D–F using anti-M6-7 antibody (kindly provided by C. Lagenaur) diluted 1∶5000, anti-CNPase antibody (Abcam, Cambridge, UK) diluted 1∶500 and anti-Neurofascin 155 antidody (Chemicon) diluted 1∶3000.

### Preparation of DRMs

Brain homogenates were extracted for 1 hour on ice in 1% Triton X-100/25 mM MES/5 mM DTT/2 mM EDTA at pH 7.0 [16] and protease inhibitors. Extracts (500 µg protein/ml buffer) were mixed with 60% Optiprep™ (Nycomed, Denmark) to reach a final concentration of 40% and overlaid in a SW40 centrifugation tube (Beckman, CA, USA) with a step gradient of 30 and 5% Optiprep™ in MES-buffer. After centrifugation at 35'000 rpm (12 hrs), 9 fractions were collected starting from the top. The raft fraction was obtained from the interphase 5–30% Optiprep™. Mouse monoclonal anti-PrP antibodies (POM-1) and mouse monoclonal anti-flotillin 2 (BD Transduction, USA) were used to characterize the Optiprep™ fractions by Western blot.

### Histopathology and Immunohistochemistry

Organs were fixed in 4% formaldehyde in PBS (pH 7.5) and paraffin-embedded. Two µm brain sections were stained with hematoxylin-eosin (HE). Immunohistochemistry was performed for glial fibrillary acidic protein (activated astrocytes) using a GFAP monoclonal antibody (DAKO, Carpinteria, CA, USA). PrP^Sc^ aggregates were detected on paraffin sections using monoclonal antibody SAF-84. For histological analyses anatomic brain regions were selected according to standard strain-typing protocols (Bruce, 1991, Fraser, 1968). Spongiosis was evaluated on a scale of 0–5 (not detectable, mild, moderate, severe, and status spongiosus). Gliosis and PrP immunoreactivity were scored on a four-degree scale (undetectable, mild, moderate, severe). Histological analyses were performed by investigators blinded to animal identification.

### Histoblot analysis

Cryosections were transferred to a nitrocellulose membrane and digested for 4 h with 20 µg/ml of proteinase K at 37°C. Blocking of the sections was done in 5% TopBlock, incubation with primary (POM1: 1∶10'000, 4A6: 1∶1000) and secondary antibodies (Dako D0486, AP goat anti mouse, 1∶1000) were done in 1% TopBlock, respectively. The blots were incubated in BCIP/NBT in B3 buffer (100 mM Tris, 100 mM NaCl, 100 mM MgCl2, pH 9.5 plus tablets and levamisole) for 45–60 min.

### Scrapie cell assay in endpoint format (SCEPA)

Prion-susceptible neuroblastoma cells (subclone N2aPK1) were exposed to 300 µl brain homogenates in 96-well plates for 3 d. Cells were subsequently split three times 1∶3 every 2 days, and three times 1∶10 every 3 days. After they reached confluence, we filtered 25,000 cells from each well onto the membrane of an ELISPOT plate, treated them with PK (0.5 µg/ml for 90 min at 37°C), denatured, and detected individual infected (PrP^Sc^-positive) cells by immunocytochemistry using alkaline phosphatase-conjugated POM1 mouse anti-PrP and an alkaline phosphatase–conjugated substrate kit (Bio-Rad). We performed serial tenfold dilutions in cell culture medium containing healthy mouse brain homogenate. Scrapie-susceptible PK1 cells were then exposed to dilutions of experimental samples ranging from 10^−4^ to 10^−9^, the same for RML, or to a 10^−4^ dilution of healthy mouse brain homogenate. Samples were quantified in endpoint format, by counting positive wells according to established methods.

### Immunoprecipitations

Brains were homogenized in 0.5% CHAPS and protease inhibitors (Complete; Roche, Switzerland) as described above. Mouse monoclonal anti-myc 4A6 antibody was cross linked to Dynabeads M-280 Sheep anti-Mouse IgG (Dynal, Norway) as recommended by the manufacturer. Four mg of total protein from 5% brain homogenates were diluted to a volume of 1.5 ml of 0.5% CHAPS/NP-40. To precipitate the PrP_myc_ complex, 40 µl of resuspended beads were added and incubated with rotational mixing for 2 hours at 4°C and for 15 min at room temperature. Beads were washed twice in PBS/0.5%CHAPS/NP-40 and twice in PBS/1% CHAPS/NP-40 at 4°C. To elute the complex, beads were incubated for 2 h at 4°C and another 10 min at room temperature with the synthetic specific peptide (c-myc: H-EQKLISEEDL-NH_2_, Roche Diagnostics, Basel, Switzerland) and the scrambled nonspecific peptide (cym: H-IELQKELDES-NH_2_, jct, Berlin, Germany) respectively. Peptides were added in 10-fold molar excess compared to the 4A6 antibody, in a final volume of 380 µl of 1% CHAPS, 1% NP-40.

### Tryptic in-gel digestion

Silver stained bands from 12% SDS PAGE were destained and incubated for 1–3 h in 100 mM ammonium bicarbonate (NH_4_HCO_3_, pH 8.0, Sigma) in 50% MeOH at 37°C. The proteins were reduced in 2 mM tris(carboxyethyl)phosphine (TCEP•HCl, Pierce, USA) in 100 mM ammonium bicarbonate at 37°C for 40 min and alkylated with 20 mM iodoacetamide (Fluka, Switzerland) for 30 min at room temperature in the dark. Gel pieces were rinsed twice in 100 mM ammonium bicarbonate, dehydrated in acetonitrile for 10 min, dried under vacuum for 10 min and reswell in 200–400 ng of sequence-grade modified trypsin solution (Promega, Madison, WI, USA) for 15 min at RT. Gel pieces were covered with sufficient amount of 100 mM ammonium bicarbonate buffer containing 2 mM CaCl_2_ and incubated overnight at 37°C. Samples were sonicated for 5 min and supernatant was pooled with an additional peptide extraction round with 50% acetonitrile/1% formic acid for 20 min at RT. Samples were dried under vacuum and kept at −20°C whenever they were not used immediately.

### ICAT labeling and sample processing

The IP eluate was precipitated by ethanol precipitation and the pellet was dissolved in 100 µl of cICAT labeling buffer (50 mM Tris, pH 8.3; 8 M Urea; 5 mM EDTA; 0.125% SDS and 0.05% RapiGest). The cICAT labeling procedures was performed as described previously [54], [55], [56]. The control sample was labeled with the light, the specific elution sample with heavy cICAT label (Applied Biosystems, Foster City, CA, USA). Digestion with trypsin (Promega, Madison, WI, USA) was performed at 37°C over night and ICAT-labeled peptides were subsequently purified according to the manufacturer's instructions. ZipTip columns (C18, Millipore, Bedford, USA) were then used for further cleanup of the affinity-purified fraction.

### Capillary chromatography and mass spectrometric analysis

Cleaned samples were resuspended in equilibration buffer (3% acetonitrile/0.1 formic acid in MilliQ-water) and loaded onto a microcapillary column constructed by slurry packing 8 cm of reversed-phase (RP) material (Magic C18, 5 µm, 200 Å, Michrom BioResources, Auburn, CA, USA) into a 75 µm fused-silica capillary (BGB Analytik AG, Böckten, Switzerland). Mass spectrometric analyses were performed on an LTQ-FT™ (Thermo Scientific, Bremen, Germany) systems directly coupled to a nanoLC™ HPLC system (eksigent, Dublin, CA, USA) at a flow rate of 200 nl/min. Peptides were eluted with an acetonitrile gradient from 3 to 45% in approximately 55 min and data-dependent acquisition of tandem mass spectra was continuously repeated during the course of the analysis. Each high accuracy MS full scan was followed by four MS/MS scans of the four most intense peaks. High mass accuracy data was search with Mascot Integra (Matrix Science, UK) using the UniProt mouse protein data base (ftp.ebi.ac.uk/pub/databases/SPproteomes/fasta/proteomes/59.M_musculus.fasta.gz), allowing for two missed trypsin cleavage sites and precursor- and fragment ion tolerances of 5 ppm and 0.8 Da, respectively. Peptides from ICAT samples were identified by searching MS/MS spectra against the same mouse protein database using Sequest [24].

PeptidePhrophet was used to assess the validity of peptide assignments. Proteins were filtered using ProteinProphet with a computed overall probability of ≥0.95 for a protein being present in the sample. Only peptide pairs that had a mass difference of 9.0301 Da were included. Both peptide contained cysteins and belonged to a protein that was identified with an Xcorr value≥1.5. Averages and standard deviations were calculated for each protein expression value when multiple peptide measurements were available. We only considered peptides with double and multiple charges, and manually evaluated the expression values by inspecting the areas of integration that the software had chosen and by adjusting them as needed. To calculate protein ratio between different pull down samples, XPRESS [56] was used.

### Prion inoculations

8–12 weeks old mice were inoculated intracerebrally (ic) or intraperitoneally (ip) with 3×10^6^ infectious units (IU) or 10×10^6^ IU, respectively, of Rocky Mountain Laboratory strain (RML, passage 5.0) brain homogenate, prepared as described [57]. Beginning 50 days after inoculation, mice were examined daily for neurological dysfunction and sacrificed on the day of onset of terminal clinical signs of scrapie. For transmission experiments, mice were inoculated ic with up 30 µl of 10% sonicated brain homogenate. Mice were monitored clinically every other day in order to ascertain the onset of clinical signs and the course of the disease. Clinical signs exacerbated over time and included progressive akinesia, priapism (males), hunchback, and stiff tail. Mice were sacrificed on the day of onset of terminal clinical signs of scrapie, defined as the time point at which they became unable to drink and/or eat.

## Supporting Information

Table S1 Inoculation of Prnp+/o and PrPmyc+/−
(0.30 MB DOC)

Table S2 Inoculation of PrPmyc−/−
(0.28 MB DOC)

Table S3 Transmission to PrPmyc+/− and PrPmyc−/−
(0.04 MB DOC)

Table S4 Proteins identified by GeLC-MS/MS after epitope elution
(0.39 MB DOC)

Table S5 Proteins with Xcorr 1.5
(0.23 MB DOC)
