# Supplementary material for: The Comprehensive Native Interactome of a Fully Functional Tagged Prion Protein
Source: PLoS One. 2009 Feb 11;4(2):e4446. doi: 10.1371/journal.pone.0004446 (PMC2635968; doi:10.1371/journal.pone.0004446)
Supplement: Table S1 — Inoculation of Prnp+/o and PrPmyc+/− (0.30 MB DOC) [file pone.0004446.s001.doc]

Table S1 Inoculation of *Prnp*^+/o^ and PrP

| Primary Inoculations; Low dose RML5 | | | | | Intracerebral Transmission to *tga20* | |
| --- | --- | --- | --- | --- | --- | --- |
| Inoculation Route | Genotype and Transgenic Line | dpi | Tissue | | PK-resistance | Incubation Time in dpi |
| ic | *Prnp*^+/o^ | 398 | |  | + |  |
| ic | *Prnp*^+/o^ | 436 | |  | + |  |
| ic | *Prnp*^+/o^ | 447 | |  | + |  |
| ic | *Tg*940 PrP | 140 | | Brain | + |  |
| ic | *Tg*940 PrP | 140 | | Brain | + |  |
| ic | *Tg*940 PrP | 250 | | Brain | + |  |
| ic | *Tg*940 PrP | 295 | | Brain | + |  |
| ic | *Tg*940 PrP | 295 | | Brain | + |  |
| ic | *Tg*940 PrP | 295 | | Brain | + | 60^#^ |
|  |  |  | |  |  | 60^#^ |
|  |  |  | |  |  | 61^#^ |
|  |  |  | |  |  | 81^#^ |
| ic | *Tg*941 PrP | 288 | | Brain | + |  |
| ic | *Tg*941 PrP | 320 | | Brain | + |  |
| ic | *Tg*941 PrP | 320 | | Brain | + |  |
| ic | *Tg*941 PrP | 336 | | Brain | + |  |
| ip | *Prnp*^+/o^ | 506 | | Brain | + |  |
| ip | *Prnp*^+/o^ | 517 | | Brain | + |  |
| ip | *Tg*940 PrP | 151 ^†^ | | Brain | n.d. |  |
| ip | *Tg*940 PrP | 193 | | Brain | + |  |
| ip | *Tg*940 PrP | 212 | | Brain | + |  |
| ip | *Tg*940 PrP | 341 | | Brain | + |  |
|  |  |  | | Spleen | + |  |
| ip | *Tg*940 PrP | 354 | | Brain | + |  |
|  |  |  | | Spleen | + |  |
|  |  | animals | | Average incubation time | Stdev. |  |
|  | *Prnp*^+/o^ ic | 3 | | 427 | 25.7 |  |
|  | *Prnp*^+/o^ ip | 2 | | 511.5 | - |  |
|  | *Tg*940 PrP ic | 6 | | 235.8 | 76.2 |  |
|  | *Tg*940 PrP ip | 5 | | 250.2 | 91.6 |  |
|  | *Tg*941 PrP ic | 4 | | 316 | 20.1 |  |
|  | | | | | | |
|  | | | | | | |
| Primary Inoculations High dose RML5 | | | | | Intracerebral Transmission to *Tg*940 PrP Mice | |
| Inoculation Route | Genotype and Transgenic Line | dpi | | Tissue | PK-resistance | Incubation Time in dpi |
| ic | *Prnp*^+/o^ | 264 | | Brain | + |  |
| ic | *Prnp*^+/o^ | 264 | | Brain | + |  |
| ic | *Prnp*^+/o^ | 281 | | Brain | + |  |
| ic | *Prnp*^+/o^ | 281 | | Brain | + |  |
| ic | *Prnp*^+/o^ | 283 | | Brain | + |  |
| ic | *Prnp*^+/o^ | 283 | | Brain | + |  |
|  |  |  | |  |  |  |
| ic | *Tg*940 PrP | 198 | | Brain | + |  |
| ic | *Tg*940 PrP | 216 | | Brain | + |  |
| ic | *Tg*940 PrP | 228 | | Brain | + | 331 |
|  |  |  | |  |  | 331 |
|  |  |  | |  |  | 331 |
|  |  |  | |  |  | 278 |
| ic | *Tg*940 PrP | 239 | | Brain | + |  |
| ic | *Tg*940 PrP | 229 | | Brain | + |  |
| ic | *Tg*940 PrP | 229 | | Brain | + |  |
| ic | *Tg*940 PrP | 233 | | Brain | + |  |
| ic | *Tg*940 PrP | 233 | | Brain | + |  |
|  |  |  | |  |  |  |
|  |  | animals | | Average incubation time | Stdev. |  |
|  | *Prnp*^+/o^ | 6 | | 276 | 9.3 |  |
|  | *Tg*940 PrP | 8 | | 225.6 | 12.9 |  |
|  |  |  | |  |  |  |
| † interpreted as intercurrent death | | | | | | |
| ^#^ killed for analysis before clinical signs | | | | | | |
| n.d. not done | | | | | | |
|  | | | | | | |
