# Supplementary material for: The Comprehensive Native Interactome of a Fully Functional Tagged Prion Protein
Source: PLoS One. 2009 Feb 11;4(2):e4446. doi: 10.1371/journal.pone.0004446 (PMC2635968; doi:10.1371/journal.pone.0004446)
Supplement: Table S2 — Inoculation of PrPmyc−/− (0.28 MB DOC) [file pone.0004446.s002.doc]

Table S2: Inoculation of PrP

| Primary Inoculations RML high dose | | | | | Intracerebral Transmission to *tga*20 |
| --- | --- | --- | --- | --- | --- |
| Inoculation Route | Genotype and Transgenic Line | dpi | Severity code* | PK-resistance | Incubation Time in dpi |
| ip | *Tg*940 PrP | 504 | 2^†^ | - |  |
| ip | *Tg*940 PrP | 77 | 2 | + |  |
| ip | *Tg*940 PrP | 660 | 3 | + |  |
| ip | *Tg*940 PrP | 621 | 2^†^ | n.d. |  |
| ip | *Tg*940 PrP | 625 | 2^†^ | n.d. |  |
| ip | *Tg*940 PrP | 725 | 2^†^ | n.d. |  |
| ip | *Tg*940 PrP | 318 | 2^†^ | n.d. |  |
| ip | *Tg*940 PrP | 730 | 3 | n.d. |  |
| ip | *Tg*940 PrP | 216 | 2^†^ | n.d. |  |
| ip | *Tg*940 PrP | 578 | 2 | n.d. |  |
| ip | *Tg*940 PrP | 150 | 2^#^ | - |  |
| ip | *Tg*940 PrP | 60 | 2^#^ | - |  |
| ip | *Tg*940 PrP | 60 | 2^#^ | - |  |
| ip | *Tg*940 PrP | 475 | 2 | - |  |
| ip | *Tg*940 PrP | 472 | 2 | weak |  |
| ip | *Tg*940 PrP | 496 | 3 | - |  |
| ip | *Tg*940 PrP | 486 | 3 | weak |  |
| ip | *Tg*940 PrP | 486 | 2 | weak |  |
| ic | *Tg*940 PrP | 61 | 2^#^ | - |  |
| ic | *Tg*940 PrP | 716 | 2^†^ | + |  |
| ic | *Tg*940 PrP | 707 | 3 | + |  |
| ic | *Tg*940 PrP | 565 | 2^†^ | n.d. |  |
| ic | *Tg*940 PrP | 140 | 2^†^ | n.d. |  |
| ip | *Tg*941 PrP | 533 | 3 | n.d. |  |
| ip | *Tg*941 PrP | 518 | 2^†^ | n.d. |  |
| ip | *Tg*941 PrP | 532 | 2^†^ | n.d. |  |
| ip | *Tg*941 PrP | 108 | 2^†^ | n.d. |  |
| ip | *Tg*941 PrP | 259 | 3 | n.d. |  |
| ip | *Tg*941 PrP | 449 | 3 | n.d. |  |
| ip | *Tg*941 PrP | 122 | 2 | n.d. |  |
| ip | *Tg*941 PrP | 178 | 2^†^ | n.d. |  |
| ic | *Tg*941 PrP | 437 | 2 | n.d. |  |
| ic | *Tg*941 PrP | 442 | 2 | n.d. |  |
| ic | *Tg*941 PrP | 442 | 2 | n.d. |  |
| ic | *Tg*941 PrP | 500 | 2^†^ | n.d. |  |
| ic | *Tg*941 PrP | 233 | 2 | - | 266^#^ |
|  |  |  |  |  | 412^#^ |
|  |  |  |  |  | 424^#^ |
|  |  |  |  |  | 432^#^ |
|  |  |  |  |  | 636 |
|  |  |  |  |  | 404^#^ |
|  |  |  |  |  | 426^#^ |
|  |  |  |  |  | 426^#^ |
|  |  |  |  |  | 519 |
|  |  |  |  |  | 519 |
| ic | *Tg*941 PrP | 495 | 2^†^ | n.d. |  |
| ic | *Tg*941 PrP | 442 | 2 | n.d. |  |
|  |  |  |  |  |  |
| * Severity code: 1: early signs, 2: clear signs and 3: terminally sick, n.d. not determined | | | | | |
| ^†^ Interpreted as intercurrent death or other physiological reasons | | | | | |
| ^#^ Killed for analysis before clinical signs | | | | | |
| n.d.: not done | | | | | |
