# Supplementary material for: The Comprehensive Native Interactome of a Fully Functional Tagged Prion Protein
Source: PLoS One. 2009 Feb 11;4(2):e4446. doi: 10.1371/journal.pone.0004446 (PMC2635968; doi:10.1371/journal.pone.0004446)
Supplement: Table S3 — Transmission to PrPmyc+/− and PrPmyc−/− (0.04 MB DOC) [file pone.0004446.s003.doc]

Table S3: Transmission to PrP and PrP

| Primary Inoculations RML high dose ic | | | | | 2^nd^ passage into PrP high dose ic | | 3^rd^ passage PrP high dose ic | |
| --- | --- | --- | --- | --- | --- | --- | --- | --- |
| Inoculation route | Genotype and transgenic line | dpi | Tissue | PK-resistance | dpi | PK-resistance | dpi | PK-resistance |
|  |  |  |  |  |  |  |  |  |
| ic | *Tg*940 PrP | 295 | brain | + |  |  |  |  |
| ic | *Tg*940 PrP | 295 | brain | + |  |  |  |  |
| ic | *Tg*940 PrP | 295 | brain | + | 525 | + |  |  |
|  |  |  |  |  | 622 | + |  |  |
|  |  |  |  |  | 622 | + | 401 | + |
|  |  |  |  |  |  |  | 310 | + |
|  |  |  |  |  |  |  | 401 | + |
|  |  |  |  |  |  |  | 361 | + |
|  |  |  |  |  |  |  | 361 | + |
| Inoculation | Average inoculation time | Stdev |  |  |  |  |  |  |
| RML | 295 | - |  |  |  |  |  |  |
| 2^nd^ passage | 589.6 | 56 |  |  |  |  |  |  |
| 3^rd^ passage | 366.8 | 37.5 |  |  |  |  |  |  |
