# Supplementary material for: The Comprehensive Native Interactome of a Fully Functional Tagged Prion Protein
Source: PLoS One. 2009 Feb 11;4(2):e4446. doi: 10.1371/journal.pone.0004446 (PMC2635968; doi:10.1371/journal.pone.0004446)
Supplement: Table S4 — Proteins identified by GeLC-MS/MS after epitope elution (0.39 MB DOC) [file pone.0004446.s004.doc]

Table S4. Proteins identified by GeLC-MS/MS after epitope elution

| **Accession Number** | **Protein Description** | **Mass (Da)** | **Number of Peptides** | **Sequence Coverage (%)** | **Mascot Protein Score** |
| --- | --- | --- | --- | --- | --- |
| P61922 | 4-aminobutyrate aminotransferase | 57100 | 2 | 7 | 153 |
| P20029 | 78 kDa glucose-regulated protein precursor | 72492 | 18 | 36 | 1256 |
| Q5SV72 | A disintegrin and metalloprotease domain 23 | 74028 | 3 | 9 | 121 |
| Q9R1V7 | ADAM 23 precursor | 94111 | 3 | 7 | 121 |
| P48962 | ADP/ATP translocase 1 (Adenine nucleotide translocator 1) | 32980 | 3 | 11 | 309 |
| P17182 | Alpha-enolase | 47322 | 4 | 14 | 270 |
| Q4LG64 | AMPA-selective glutamate receptor 2 flip type | 99411 | 4 | 8 | 370 |
| P17426 | AP-2 complex subunit alpha-1 | 108679 | 4 | 7 | 259 |
| P08226 | Apolipoprotein E precursor (Apo-E) | 35901 | 4 | 14 | 206 |
| Q03265 | ATP synthase subunit alpha, mitochondrial precursor | 59830 | 8 | 22 | 396 |
| P18572 | Basigin precursor | 42874 | 2 | 6 | 110 |
| Q14CE2 | Bcas1 protein | 62642 | 3 | 8 | 133 |
| Q9Z2V0 | Beta spectrin homolog | 25628 | 2 | 9 | 109 |
| Q91XV3 | Brain acid soluble protein 1 | 21943 | 10 | 72 | 828 |
| Q80YN3 | Breast carcinoma amplified sequence 1 homolog | 67874 | 2 | 5 | 125 |
| Q9Z1L5 | Calcium channel alpha-2-delta-C subunit | 123954 | 12 | 15 | 865 |
| P35564 | Calnexin precursor | 67635 | 2 | 4 | 133 |
| P31324 | cAMP-dependent protein kinase type II-beta | 46406 | 5 | 17 | 380 |
| Q80V42 | Carboxypeptidase M precursor | 50923 | 4 | 10 | 211 |
| Q60849 | CD98 heavy chain | 58899 | 4 | 9 | 243 |
| Q6X893 | Choline transporter-like protein 1 | 74574 | 4 | 9 | 287 |
| Q4VA91 | Chondroitin sulfate proteoglycan 2 | 41758 | 3 | 12 | 266 |
| Q68FD5 | Clathrin heavy chain | 193202 | 58 | 43 | 4731 |
| P16330 | CNP, 2",3"-cyclic-nucleotide 3"-phosphodiesterase | 47493 | 13 | 42 | 778 |
| P12960 | Contactin-1 precursor (Neural cell surface protein F3) | 114172 | 36 | 45 | 3071 |
| P00405 | Cytochrome c oxidase subunit 2 | 26130 | 2 | 13 | 151 |
| O08532 | Dihydropyridine-sensitive L-type calcium channel | 125692 | 23 | 27 | 1565 |
| P39053 | Dynamin-1 | 98140 | 6 | 10 | 324 |
| Q8BGN3 | Ectonucleotide pyrophosphatase/phosphodiesterase | 50928 | 6 | 22 | 552 |
| O88343 | Electrogenic sodium bicarbonate cotransporter 1 | 122318 | 2 | 3 | 145 |
| Q99LC5 | Electron transfer flavoprotein subunit alpha | 35360 | 2 | 13 | 156 |
| P62631 | Elongation factor 1-alpha 2 | 50764 | 4 | 12 | 224 |
| Q6PHC1 | Eno1 protein | 40099 | 2 | 8 | 137 |
| Q922A0 | Eno2 protein | 37408 | 3 | 14 | 238 |
| Q9Z0N1 | Eukaryotic translation initiation factor 2 | 51472 | 7 | 25 | 390 |
| P47754 | F-actin capping protein alpha-2 subunit | 32987 | 2 | 9 | 115 |
| P17183 | Gamma-enolase | 47478 | 4 | 17 | 296 |
| P06745 | Glucose-6-phosphate isomerase | 62825 | 2 | 6 | 176 |
| P23819 | Glutamate receptor 2 precursor | 99226 | 4 | 8 | 370 |
| P16858 | Glyceraldehyde-3-phosphate dehydrogenase | 35941 | 8 | 37 | 592 |
| Q8BG05 | Heterogeneous nuclear ribonucleoprotein | 39856 | 7 | 20 | 479 |
| P17710 | Hexokinase-1 | 109544 | 7 | 9 | 310 |
| Q9QUP5 | Hyaluronan and proteoglycan link protein 1 | 41079 | 7 | 31 | 444 |
| Q99LC4 | Igh-4 protein | 51659 | 3 | 13 | 242 |
| Q9D8B7 | Junctional adhesion molecule C precursor (JAM-C) | 35386 | 3 | 11 | 141 |
| Q61735 | Leukocyte surface antigen CD47 precursor | 33589 | 3 | 11 | 357 |
| Q8BLK3 | Limbic system-associated membrane protein precursor | 38462 | 14 | 44 | 1071 |
| P16125 | L-lactate dehydrogenase B chain | 36703 | 4 | 14 | 191 |
| P32067 | Lupus La protein homolog | 47898 | 2 | 9 | 140 |
| P04925 | Major prion protein precursor (PrP) (PrP27-30) | 28131 | 4 | 17 | 221 |
| P14152 | Malate dehydrogenase, cytoplasmic | 36528 | 5 | 20 | 285 |
| Q9QYS2 | Metabotropic glutamate receptor 3 precursor | 100932 | 2 | 4 | 112 |
| P14873 | Microtubule-associated protein 1B (MAP 1B) | 271556 | 5 | 3 | 353 |
| P20917 | Myelin-associated glycoprotein precursor (Siglec-4a) | 70071 | 2 | 3 | 128 |
| Q8VDQ8 | NAD-dependent deacetylase sirtuin-2 | 43856 | 4 | 15 | 185 |
| P13595 | Neural cell adhesion molecule (NCAM-180) | 120076 | 12 | 19 | 1176 |
| P55066 | Neurocan core protein precursor | 139053 | 4 | 4 | 147 |
| Q810U3 | Neurofascin precursor | 138688 | 9 | 12 | 766 |
| P06837 | Neuromodulin (Axonal membrane protein GAP-43) | 23732 | 6 | 43 | 425 |
| Q80Z24 | Neuronal growth regulator 1 precursor | 38560 | 11 | 40 | 754 |
| P35802 | Neuronal membrane glycoprotein M6-a (M6a) | 31870 | 5 | 14 | 499 |
| P35803 | Neuronal membrane glycoprotein M6-b (M6b) | 36984 | 3 | 10 | 184 |
| Q99PJ0 | Neurotrimin precursor | 38531 | 14 | 42 | 1250 |
| O35874 | Neutral amino acid transporter A (SATT) | 56311 | 3 | 11 | 178 |
| Q6GQT9 | Nodal modulator 1 | 134362 | 3 | 3 | 222 |
| Q9QWJ7 | Non-erythrocyte beta spectrin | 251686 | 19 | 12 | 1312 |
| P09405 | Nucleolin (Protein C23) | 76603 | 12 | 16 | 703 |
| Q63912 | Oligodendrocyte-myelin glycoprotein precursor | 49708 | 3 | 13 | 174 |
| Q9Z0P4 | Paralemmin | 41760 | 2 | 9 | 145 |
| Q9R0K7 | Plasma membrane calcium-transporting ATPase 2 | 133701 | 2 | 3 | 219 |
| Q62029 | PolyA binding protein, testis-enriched isoform | 69474 | 4 | 11 | 358 |
| P29341 | Polyadenylate-binding protein 1 | 70826 | 8 | 15 | 647 |
| Q9EQU5 | Protein SET (Phosphatase 2A inhibitor I2PP2A) | 33358 | 3 | 13 | 216 |
| P52480 | Pyruvate kinase isozyme M2 | 58289 | 6 | 16 | 325 |
| P97379 | Ras-GTPase-activating protein-binding protein 2 | 54112 | 4 | 13 | 286 |
| Q20BD0 | S1 protein C2 | 36302 | 4 | 17 | 238 |
| Q8K021 | Secretory carrier-associated membrane protein 1 | 38403 | 2 | 10 | 113 |
| P63328 | Serine/threonine-protein phosphatase | 59291 | 6 | 13 | 346 |
| P31650 | Sodium- and chloride-dependent GABA transporter | 71268 | 3 | 7 | 315 |
| Q8K596 | Sodium-calcium exchanger | 101788 | 3 | 5 | 249 |
| P16546 | Spectrin alpha chain, brain | 168020 | 26 | 27 | 1724 |
| O55129 | STOP protein | 96690 | 5 | 7 | 194 |
| P38647 | Stress-70 protein, mitochondrial precursor | 73768 | 14 | 28 | 907 |
| Q8BG39 | Synaptic vesicle glycoprotein 2B | 78204 | 3 | 5 | 182 |
| Q62277 | Synaptophysin (Major synaptic vesicle protein p38) | 34288 | 5 | 15 | 178 |
| P60879 | Synaptosomal-associated protein 25 (SNAP-25) | 23528 | 2 | 12 | 283 |
| P46096 | Synaptotagmin-1 (Synaptotagmin I) | 47730 | 17 | 42 | 1470 |
| Q8BYI9 | Tenascin-R precursor (TN-R) | 151807 | 9 | 9 | 736 |
| P42669 | Transcriptional activator protein Pur-alpha | 34976 | 6 | 26 | 415 |
| Q01853 | Transitional endoplasmic reticulum ATPase | 89805 | 3 | 7 | 133 |
| P68369 | Tubulin alpha-1 chain | 50788 | 7 | 28 | 645 |
| Q9DB77 | Ubiquinol-cytochrome-c reductase complex core protein 2 | 48262 | 2 | 8 | 132 |
| P51863 | Vacuolar ATP synthase subunit d | 40731 | 8 | 27 | 311 |
| Q62059 | Versican core protein precursor | 368613 | 4 | 2 | 278 |
| O35633 | Vesicular inhibitory amino acid transporter | 58313 | 5 | 10 | 331 |
| Q6REE3 | Voltage-gated calcium channel alpha2-delta2 subunit | 135362 | 17 | 15 | 873 |
