# Supplementary material for: The Comprehensive Native Interactome of a Fully Functional Tagged Prion Protein
Source: PLoS One. 2009 Feb 11;4(2):e4446. doi: 10.1371/journal.pone.0004446 (PMC2635968; doi:10.1371/journal.pone.0004446)
Supplement: Table S5 — Proteins with Xcorr 1.5 (0.23 MB DOC) [file pone.0004446.s005.doc]

Table S5: Proteins with Xcorr 1.5

| Entrez_ID | Mass (Da) | Ratio | Protein name |
| --- | --- | --- | --- |
| AN32A_MOUSE | 11737 | 1:0.06 | ACIDIC (LEUCINE-RICH) NUCLEAR PHOSPHOPROTEIN 32 FAMILY |
| ACTN1_MOUSE | 109711 | 1:0.38 | ACTININ, ALPHA 1 |
| ACSL6_MOUSE | 216739 | 1:0.11 | ACYL-COA SYNTHETASE LONG-CHAIN FAMILY MEMBER 6 |
| AP1B1_MOUSE | 11764 | 1:0.25 | ADAPTOR PROTEIN COMPLEX AP-1, BETA 1 SUBUNIT |
| AP2A1_MOUSE | 11771 | 1:0.11 | ADAPTOR PROTEIN COMPLEX AP-2, ALPHA 1 SUBUNIT |
| AP2M1_MOUSE | 11773 | 1:0.01 | ADAPTOR PROTEIN COMPLEX AP-2, MU1 |
| ANK1_MOUSE | 11733 | 1:0.48 | ANKYRIN 1, ERYTHROID |
| AT2A2_MOUSE | 11938 | 1:0.03 | ATPASE, CA++ TRANSPORTING, CARDIAC MUSCLE, SLOW TWITCH 2 |
| AT2B2_MOUSE | 11941 | 1:0.06 | ATPASE, CA++ TRANSPORTING, PLASMA MEMBRANE 2 |
| VPP1_MOUSE | 11975 | 1:0.40 | ATPASE, H+ TRANSPORTING, LYSOSOMAL V0 SUBUNIT A1 |
| VA0D_MOUSE | 11972 | 1:0.25 | ATPASE, H+ TRANSPORTING, LYSOSOMAL V0 SUBUNIT D1 |
| VATB2_MOUSE | 11966 | 1:0.17 | ATPASE, H+ TRANSPORTING, LYSOSOMAL V1 SUBUNIT B2 |
| AT12A_MOUSE | 192113 | 1:0.05 | ATPASE, H+/K+ TRANSPORTING, NONGASTRIC, ALPHA POLYPEPTIDE |
| AT1A1_MOUSE | 11928 | 1:0.06 | ATPASE, NA+/K+ TRANSPORTING, ALPHA 1 POLYPEPTIDE |
| Q6ZQ49 | 98660 | 1:0.02 | ATPASE, NA+/K+ TRANSPORTING, ALPHA 2 POLYPEPTIDE |
| AT1A3_MOUSE | 232975 | 1:0.3 | ATPASE, NA+/K+ TRANSPORTING, ALPHA 3 POLYPEPTIDE |
| AT1B1_MOUSE | 11931 | 1:0.13 | ATPASE, NA+/K+ TRANSPORTING, BETA 1 POLYPEPTIDE |
| PGCB_MOUSE | 12032 | 1:0.17 | BREVICAN |
| BIN1_MOUSE | 30948 | 0.86:1 | BRIDGING INTEGRATOR 1 |
| KCC2B_MOUSE | 12323 | 1:0.07 | CALCIUM/CALMODULIN-DEPENDENT PROTEIN KINASE II, BETA |
| TCPE_MOUSE | 12465 | 1:0.71 | CHAPERONIN SUBUNIT 5 (EPSILON) |
| CSPG2_MOUSE | 13003 | 1:0.17 | CHONDROITIN SULFATE PROTEOGLYCAN 2 |
| CSPG3_MOUSE | 13004 | 1:0.46 | CHONDROITIN SULFATE PROTEOGLYCAN 3 |
| CISY_MOUSE | 12974 | 1:0.09 | CITRATE SYNTHASE |
| Q80U89 | 67300 | 1:0.23 | CLATHRIN, HEAVY POLYPEPTIDE (HC) |
| COF1_MOUSE | 12631 | 1:0.80 | COFILIN 1, NON-MUSCLE |
| CNTN1_MOUSE | 12805 | 1:0.28 | CONTACTIN 1 |
| COR1B_MOUSE | 23789 | 1:0.27 | CORONIN, ACTIN BINDING PROTEIN 1B |
| COR2B_MOUSE | 235431 | 1:0.51 | CORONIN, ACTIN BINDING PROTEIN, 2B |
| SRC8_MOUSE | 13043 | 0.08:1 | CORTACTIN |
| KCRU_MOUSE | 12716 | 0:016 | CREATINE KINASE, MITOCHONDRIAL 1, UBIQUITOUS |
| CN37_MOUSE | 12799 | 1:0.15 | CYCLIC NUCLEOTIDE PHOSPHODIESTERASE 1 |
| COX5A_MOUSE | 12858 | 1:0.72 | CYTOCHROME C OXIDASE, SUBUNIT VA |
| DPYL2_MOUSE | 12934 | 1:0.05 | DIHYDROPYRIMIDINASE-LIKE 2 |
| Q99LF4 | 28088 | 1:0.53 | DNA SEGMENT, CHR 10, WAYNE STATE UNIVERSITY 52, EXPRESSED |
| DYN1_MOUSE | 13429 | 0.58:1 | DYNAMIN 1 |
| E41L3_MOUSE | 13823 | 1:0.03 | ERYTHROCYTE PROTEIN BAND 4.1-LIKE 3 |
| IF5A_MOUSE | 276770 | 1:0.51 | EUKARYOTIC TRANSLATION INITIATION FACTOR 5A |
| Q8C445 | 109676 | 1:0.24 | EXPRESSED SEQUENCE AI835472 |
| SND1_MOUSE | 56463 | 1:0.13 | EXPRESSED SEQUENCE AL033314 |
| FAS_MOUSE | 14104 | 1:0.01 | FATTY ACID SYNTHASE |
| FBX2_MOUSE | 230904 | 1:0.71 | F-BOX ONLY PROTEIN 2 |
| CXA1_MOUSE | 14609 | 1:0.11 | GAP JUNCTION MEMBRANE CHANNEL PROTEIN ALPHA 1 |
| Q8BUV3 | 268566 | 1:0.08 | GEPHYRIN |
| DHE3_MOUSE | 14661 | 1:0.14 | GLUTAMATE DEHYDROGENASE 1 |
| GLNA_MOUSE | 14645 | 1:0.28 | GLUTAMATE-AMMONIA LIGASE (GLUTAMINE SYNTHETASE) |
| GPM6A_MOUSE | 234267 | 1:0.11 | GLYCOPROTEIN M6A |
| GNAO1_MOUSE | 14681 | 1:0.12 | GUANINE NUCLEOTIDE BINDING PROTEIN, ALPHA O |
| GBB2_MOUSE | 14693 | 1:0.06 | GUANINE NUCLEOTIDE BINDING PROTEIN, BETA 2 |
| HS70A_MOUSE | 193740 | 1:0.01 | HEAT SHOCK PROTEIN 1B |
| Q8BXY2 | 209462 | 0.35:1 | HECT DOMAIN AND ANKYRIN REPEAT CONTAINING |
| HDGF_MOUSE | 15191 | 0.29:1 | HEPATOMA-DERIVED GROWTH FACTOR |
| HCN2_MOUSE | 15166 | 1:0.26 | HYPERPOLARIZATION-ACTIVATED, CYCLIC NUCLEOTIDE-GATED K+ 2 |
| Q7TNR6 | 230868 | 1:0.30 | IMMUNOGLOBIN SUPERFAMILY, MEMBER 21 |
| ICAM5_MOUSE | 15898 | 1:0.26 | INTERCELLULAR ADHESION MOLECULE 5, TELENCEPHALIN |
| IRF2_MOUSE | 16363 | 1:0.17 | INTERFERON REGULATORY FACTOR 2 |
| AINX_MOUSE | 226180 | 1:0.02 | INTERNEXIN NEURONAL INTERMEDIATE FILAMENT PROTEIN, ALPHA |
| MAP2_MOUSE | 17756 | 1:0.13 | MICROTUBULE-ASSOCIATED PROTEIN 2 |
| MOG_MOUSE | 17441 | 1:0.28 | MYELIN OLIGODENDROCYTE GLYCOPROTEIN |
| MTMR1_MOUSE | 53332 | 1:0.11 | MYOTUBULARIN RELATED PROTEIN 1 |
| NUAM_MOUSE | 227197 | 1:0.30 | NADH DEHYDROGENASE (UBIQUINONE) FE-S PROTEIN 1 |
| Q5M9J7 | 407785 | 1:0.09 | NADH DEHYDROGENASE (UBIQUINONE) FE-S PROTEIN 6 |
| NCAM2_MOUSE | 17968 | 1:0.34 | NEURAL CELL ADHESION MOLECULE 2 |
| NFASC_MOUSE | 269116 | 1:0.12 | NEUROFASCIN |
| OPA1_MOUSE | 74143 | 1:0.20 | OPTIC ATROPHY 1 HOMOLOG (HUMAN) |
| ODO1_MOUSE | 18293 | 1:0.41 | OXOGLUTARATE DEHYDROGENASE (LIPOAMIDE) |
| LPP3_MOUSE | 67916 | 1:0.02 | PHOSPHATIDIC ACID PHOSPHATASE TYPE 2B |
| PHYIP_MOUSE | 105653 | 1:0.19 | PHYTANOYL-COA HYDROXYLASE INTERACTING PROTEIN |
| PRIO_MOUSE | 19122 | 1:0.07 | PRION PROTEIN |
| KPCB_MOUSE | 18751 | 1:0.28 | PROTEIN KINASE C, BETA 1 |
| IPPD_MOUSE | 19049 | 1:0.15 | PROTEIN PHOSPHATASE 1, REGULATORY (INHIBITOR) SUBUNIT 1B |
| MYPR_MOUSE | 18823 | 1:0.06 | PROTEOLIPID PROTEIN (MYELIN) 1 |
| ODPA_MOUSE | 18597 | 1:0.41 | PYRUVATE DEHYDROGENASE E1 ALPHA 1 |
| Q80TQ6 | 98732 | 1:0.32 | RAB3 GTPASE ACTIVATING PROTEIN SUBUNIT 2 |
| Q6A063 | 110351 | 0.04:1 | RAP1, GTPASE-ACTIVATING PROTEIN 1 |
| RL11_MOUSE | 67025 | 1:0.04 | RIBOSOMAL PROTEIN L11 |
| RL12_MOUSE | 269261 | 1:0.06 | RIBOSOMAL PROTEIN L12 |
| RL18A_MOUSE | 76808 | 1:0.14 | RIBOSOMAL PROTEIN L18A |
| RS16_MOUSE | 20055 | 1:0.01 | RIBOSOMAL PROTEIN S16 |
| RS27L_MOUSE | 67941 | 1:0.02 | RIBOSOMAL PROTEIN S27-LIKE |
| RS5_MOUSE | 20103 | 1:0.05 | RIBOSOMAL PROTEIN S5 |
| FTHFD_MOUSE | 107747 | 1:0.09 | RIKEN CDNA 1810048F20 GENE |
| ROA3_MOUSE | 229279 | 1:0.47 | RIKEN CDNA 2610510D13 GENE |
| NRCAM_MOUSE | 319504 | 1:0.08 | RIKEN CDNA C030017F07 GENE |
| SALL2_MOUSE | 50524 | 1:0.51 | SAL-LIKE 2 (DROSOPHILA) |
| SCRN1_MOUSE | 69938 | 1:0.17 | SECERNIN 1 |
| SEPT5_MOUSE | 18951 | 1:0.41 | SEPTIN 5 |
| SHLB2_MOUSE | 227700 | 1:0.02 | SH3-DOMAIN GRB2-LIKE ENDOPHILIN B2 |
| EAA2_MOUSE | 20511 | 1:0.05 | SOLUTE CARRIER FAMILY 1 (GLIAL GLUTAMATE TRANSPORTER 2) |
| EAA1_MOUSE | 20512 | 1:0.07 | SOLUTE CARRIER FAMILY 1 (GLIAL GLUTAMATE TRANSPORTER 3) |
| S12A5_MOUSE | 57138 | 1:0.02 | SOLUTE CARRIER FAMILY 12, MEMBER 5 |
| S12A6_MOUSE | 107723 | 1:0.13 | SOLUTE CARRIER FAMILY 12, MEMBER 6 |
| ADT2_MOUSE | 11740 | 0.17:1 | MITOCHONDRIAL CARRIER, ADENINE NUCLEOTIDE TRANSLOCATOR |
| SPTA2_MOUSE | 20740 | 1:0.19 | SPECTRIN ALPHA 2 |
| SPTB2_MOUSE | 20742 | 0.22:1 | SPECTRIN BETA 2 |
| Q68FG2 | 20743 | 1:0.28 | SPECTRIN BETA 3 |
| STRN4_MOUSE | 97387 | 1:0.47 | STRIATIN, CALMODULIN BINDING PROTEIN 4 |
| Q9QWV7 | 20965 | 1:0.41 | SYNAPSIN II |
| AP180_MOUSE | 20616 | 1:0.27 | SYNAPTOSOMAL-ASSOCIATED PROTEIN 91 |
| Q71LX4 | 70549 | 1:0.06 | TALIN 2 |
| TBA1_MOUSE | 22142 | 1:0.57 | TUBULIN, ALPHA 1 |
| TBA2_MOUSE | 22143 | 1:0-03 | TUBULIN, ALPHA 2 |
| TBB3_MOUSE | 22152 | 1:0.04 | TUBULIN, BETA 3 |
| 1433Z_MOUSE | 22631 | 1:0.24 | TYROSINE 3-MONOOXYGENASE/TRYPTOPHAN 5-MONOOXYGENASE |
| UQCR1_MOUSE | 22273 | 1:0.49 | UBIQUINOL-CYTOCHROME C REDUCTASE CORE PROTEIN 1 |
| UCRH_MOUSE | 66576 | 1:0.49 | UBIQUINOL-CYTOCHROME C REDUCTASE HINGE PROTEIN |
| TERA_MOUSE | 269523 | 1:0.07 | VALOSIN CONTAINING PROTEIN |
| VATE_MOUSE | 11973 | 1:0.17 | VATPASE, H+ TRANSPORTING, LYSOSOMAL V1 SUBUNIT E1 |
| VDAC1_MOUSE | 22333 | 1:0.06 | VOLTAGE-DEPENDENT ANION CHANNEL 1 |
| VDAC3_MOUSE | 22335 | 1:0.35 | VOLTAGE-DEPENDENT ANION CHANNEL 3 |
|  |  |  |  |
